# Impaired Efferocytosis Enables Apoptotic Osteoblasts to Escape Osteoimmune Surveillance During Aging

**DOI:** 10.1002/advs.202303946

**Published:** 2023-10-28

**Authors:** Rongyao Xu, Hanyu Xie, Xin Shen, Jiadong Huang, Hengguo Zhang, Yu Fu, Ping Zhang, Songsong Guo, Dongmiao Wang, Sheng Li, Kai Zheng, Wen Sun, Laikui Liu, Jie Cheng, Hongbing Jiang

**Affiliations:** ^1^ Jiangsu Key Laboratory of Oral Diseases Nanjing Medical University Nanjing Jiangsu Province 210029 China; ^2^ Department of Oral and Maxillofacial Surgery Affiliated Hospital of Stomatology Nanjing Medical University Nanjing Jiangsu Province 210029 China; ^3^ Jiangsu Province Engineering Research Center of Stomatological Translational Medicine Nanjing Jiangsu Province 210029 China; ^4^ Department of Basic Science of Stomatology Affiliated Hospital of Stomatology Nanjing Medical University Nanjing Jiangsu 211166 China

**Keywords:** aging, CD47, checkpoint, efferocytosis, osteoimmune, SIRT6

## Abstract

Macrophage efferocytosis of apoptotic osteoblasts (apoOBs) is a key osteoimmune process for bone homeostasis. However, apoOBs frequently accumulate in aged bone marrow, where they may mount proinflammatory responses and progressive bone loss. The reason why apoOBs are not cleared during aging remains unclear. In this study, it is demonstrated that aged apoOBs upregulate the immune checkpoint molecule CD47, which is controlled by SIRT6‐regulated transcriptional pausing, to evade clearance by macrophages. Using osteoblast‐ and myeloid‐specific gene knockout mice, SIRT6 is further revealed to be a critical modulator for apoOBs clearance via targeting CD47‐SIRPα checkpoint. Moreover, apoOBs activate SIRT6‐mediated chemotaxis to recruit macrophages by releasing apoptotic vesicles. Two targeting delivery strategies are developed to enhance SIRT6 activity, resulting in rejuvenated apoOBs clearance and delayed age‐related bone loss. Collectively, the findings reveal a previously unknown linkage between immune surveillance and bone homeostasis and targeting the SIRT6‐regulated mechanism can be a promising therapeutic strategy for age‐related bone diseases.

## Graphical abstract

Aging‐induced immunosenescence is characterized as impaired immune surveillance, contributing to many age‐related diseases. However, there is little investigation about how immune surveillance regulates bone homeostasis during aging. Here, for the first time, a new concept of osteoimmune surveillance is put forward and link it to impaired macrophage efferocytosis of apoptotic osteoblasts due to dysfunctional CD47–SIRPα checkpoint.

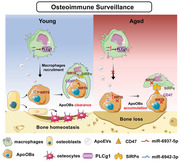

## 1 Introduction

Age‐associated dysfunctional immune response, also known as immunosenescence, lose their ability to respond to various environmental and endogenous stress, driving most chronic diseases owing to blunted immune surveillance.^[^ ^1^, ^2^ ^]^ Although age‐related bone loss has been well recognized as an imbalanced homeostasis of osteoblast‐osteoclast coupling, whether and how aging participates in regulating immune surveillance in bone (termed “osteoimmune surveillance”) remains largely unexplored. Osteoimmune surveillance focuses on the connection of the bone and immune system. It is critical for skeletal development, bone homeostasis, and regeneration through activating various cell types, signaling pathways, and cytokines.^[^ ^3^, ^4^, ^5^ ^]^ One such process is a process called efferocytosis, i.e., the swift removal of apoptotic osteoblasts (apoOBs) by macrophages that maintain bone homeostasis by recruiting and giving way to new osteoblasts.^[^ ^6^, ^7^, ^8^ ^]^ Considering the observation of increased apoOBs accumulation in the aged bone marrow microenvironment in our study or previous studies, we hoped to explore whether apoOBs would evade osteoimmune surveillance during aging, thus leading to progressive bone loss, and whether these relationships could be targeted therapeutically.^[^ ^9^, ^10^, ^11^ ^]^

Immunosenescence is characterized by changes in age‐related biomarkers of many functional cells, such as the expressing p16 or the production of senescence‐associated secretory phenotype (SASP).^[^ ^12^, ^13^, ^14^ ^]^ These accumulated senescent cells contribute to a proinflammatory tissue state and an increase in disease susceptibility, leading to mortality in older adults.^[^ ^15^ ^]^ It is worth noting that clearance of senescent cells can indeed rejuvenate aging phenotypes.^[^ ^16^ ^]^ Recently, Wang et al. reported that programmed death‐ligand 1 (PD‐L1, an immune checkpoint protein)‐positive senescent cells were resistant to T cell surveillance in naturally aging mice; however, blocking this checkpoint reduced the accumulation of senescent cells and ameliorated inflammation associated with aging.^[^ ^1^ ^]^ Another immune checkpoint molecule, CD47, which is widely expressed in a variety of cancers, which renders tumor cells resistant to immune surveillance upon binding to the signal‐regulatory protein alpha (SIRPα) receptor on macrophages.^[^ ^17^, ^18^ ^]^ Evidence has reported that upregulation of CD47 occurs with aging in muscle stem cells or red blood cells and importantly, CD47‐mediated efferocytosis is a commonly physiological mechanism by which cells regulate their phagocytosis in atherosclerosis prevention and cancer therapy.^[^ ^19^, ^20^, ^21^, ^22^, ^23^ ^]^ However, there has been no evidence so far of a direct linkage between the CD47‐SIRPa checkpoint and the regulation of apoOBs efferocytosis in the context of age‐related bone loss.

The pathogenesis of age‐related disease is strongly correlated with dysregulated nicotinamide adenine dinucleotide (NAD^+^) metabolism, whereas restoration of NAD^+^ production enabled the prolonged healthspan and lifespan in a sirtuin‐dependent manner.^[^ ^24^, ^25^ ^]^ As NAD^+^‐dependent deacetylases, the sirtuin family of proteins (SIRT1‐7) are critical in maintaining normal bone homeostasis, but their dysregulation on bone cells with aging might contribute to the progressive bone loss.^[^ ^26^ ^]^ Among these sirtuins, SIRT6 functions at the level of chromatin to widely silence gene transcription through deacetylation of H3K9ac, H3K18ac, and H3K56ac, which is indispensable for skeletal development and bone homeostasis.^[^ ^27^, ^28^ ^]^ Although SIRT6 is capable of alleviating hypoxia‐induced apoptosis of osteoblasts, it remains unknown whether SIRT6 is responsible for the clearance of apoptotic osteoblasts in pathophysiological conditions, whereas reduced expression is associated with accumulated apoOBs in the aged bone marrow microenvironment.^[^ ^29^ ^]^

Here, we found that accumulated apoOBs in aged mice that were difficult to be efferocytosed by macrophages displayed increased expression of CD47, whereas blockage of CD47 with antibodies promoted apoOBs clearance and delayed age‐related bone loss. To avoid the probable side‐effect of CD47 blockade‐induced hematotoxicity, we further explored the upstream regulatory mechanism and identified SIRT6 as a key mediator of CD47‐SIRPα checkpoint to eliminate apoOBs. Subsequently, its function was further verified in vivo by constructing osteoblast‐ and myeloid‐specific SIRT6 knockout mice. Additionally, we also found that apoOBs could regulate macrophage migration via releasing miRNAs‐loaded apoptotic vesicles (apoVs) in a SIRT6‐dependent manner. Finally, two therapeutical strategies were designed to enhance SIRT6 expression, contributing to rejuvenated apoOBs clearance and delayed bone loss in aged bone. In summary, we unraveled a previously unknown linkage between immune surveillance and bone homeostasis.

## 2 Results

### 2.1 Increased CD47 Expression in Accumulated apoOBs from Aged Bone Marrow

**Figure 1:**
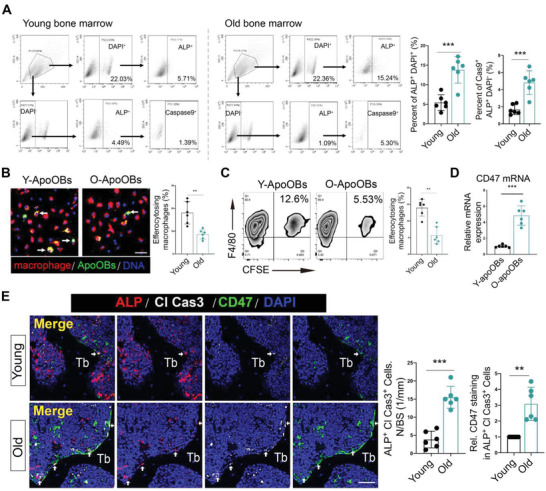
Accumulated apoOBs in old mice are highly expressed CD47 and associated with bone loss. A) The gating strategy used to isolate and compare ALP^+^ DAPI^+^ cells and ALP^+^ Caspase9^+^ DAPI^−^ cells in young and aged bone marrow. The right panel showed the quantitative data (*n* = 6). B) OBs stained with CFSE were induced to apoptosis and cocultured with macrophage in equal number (1:1) for 3 h. Macrophages were indicated by immunofluorescence staining using F4/80 antibodies. Arrow, phagocytosed cell. The right panel showed the quantitative data (*n* = 6). Scale bar = 100 µm. C) Efferocytic macrophages were then examined for flow cytometric analysis. The right panel showed the quantitative data (*n* = 6). D) Quantitative qRT‐PCR analysis of the mRNA levels of CD47 in young and old apoOBs (*n* = 6). E) Representative images of ALP, cleaved caspase3 and CD47 immunostaining in young and old bone tissue. White arrow, CD47 in apoOBs. Tb, trabecular bone. The right panel showed the quantitative measurements of apoOBs (ALP^+^/Cl caspase3^+^ cells) and CD47 in apoOBs on the bone surface (*n* = 6). Scale bar = 25 µm. Results are presented as the mean ± S.D. **p < 0.01; ***p < 0.001 by unpaired 2‐tailed Student's *t*‐test.

To first explore whether apoOBs would be accumulated in the course of aging, fresh young, and aged bone marrow were isolated for intracellular flow cytometry detection and stained with DAPI and ALP to select the live/dead cells and osteoblasts, respectively. We found that the proportion of both ALP^+^ DAPI^+^ cells and ALP^+^ Caspase9^+^ DAPI^−^ cells were indeed higher in aged bone marrow than that in young bone marrow (**Figure** **1**A). We next sought to ascertain age‐dependent accumulation of apoOBs at single‐cell resolution. Thus, a public scRNA‐seq dataset from a recent study using LepR‐Cre‐traced cells to label most bone marrow stromal and osteogenic lineage cells (OLCs) was reanalyzed.^[^ ^30^ ^]^ A total of 5022 cells from young mice and 2619 cells from aged mice were obtained for t‐distributed stochastic neighbor embedding (t‐SNE) to identify OLCs (Figure S1A, Supporting Information). Then, The OLCs were sub‐divided into five subsets (Figure S1B and Table S1, Supporting Information), including Pre‐OBs (cluster 0,1, expressing Cxcl12, ApoE, LepR, and Grem1), OBs (cluster 2, expressing CD200, Postn, Spp1, and Alpl), apoOBs (cluster 3, highly expressing mitochondrial genes), and osteocytes (cluster 4, expressing Dmp1, and Col1a1).^[^ ^30^, ^31^, ^32^, ^33^, ^34^, ^35^, ^36^, ^37^ ^]^ We mapped the differentiation trajectories of OLCs using pseudotemporal cell trajectory analysis and observed that one branch where most of apoOBs located were split out during the differentiation of OBs into osteocytes (Figure S1C, Supporting Information). By comparing the proportion of different clusters in young and aged OLCs, we found that the proportion of osteocytes was obviously decreased upon aging, while the proportion of pre‐OBs (cluster 0), pre‐OBs (cluster 1), or apoOBs was nearly unchanged (Figure S1D, Supporting Information), probably due to the initial removal of a subset of apoptotic cells during the pre‐processing of scRNA‐seq data. Although age‐dependent apoOBs accumulation could not be demonstrated by scRNA‐seq analysis, these results indicated a trend of apoOBs during the process of osteoblasts to osteocytes.

Next, to ascertain whether apoOBs accumulation in aged bone marrow is associated with the impaired efferocytosis, primary osteoblasts were cultured from young (3 months age) and old mice (15 months age) were cultured. Our findings revealed that without apoptotic induction, neither young nor aged OBs could be efferocytosed by macrophages (Figure S2A, Supporting Information), while apoOBs were expectedly engulfed by macrophages (Figure 1B,C). This suggests that apoptotic induction in vitro is necessary to initiate efferocytosis. Moreover, apoOBs from old mice were more difficult to be efferocytosed by macrophages than those from young group. To explore the reason for this dysregulation of impaired efferocytosis, apoOBs isolated from young and old mice were subjected to RNA sequencing (RNA‐seq). As mentioned above, CD47 is capable of regulating efferocytosis; interestingly, CD47 was markedly increased (*p* = 1.91   × 10^−5^) in aged apoOBs (Figure S2B and Table S2, Supporting Information).^[^ ^22^ ^]^ Highly expressed CD47 expression in aged apoOBs was further confirmed by qPCR and immunofluorescence analyses (Figure 1D; and Figure S2C, Supporting Information). Intracellular flow cytometry also revealed that although total ALP^+^ cells decreased in the aged bone marrow, the percentage of CD47^+^ apoOBs (ALP^+^ caspase9^+^ cells) was higher than that in the young group (Figure S2D, Supporting Information). To replicate these in vitro results, old C57/B6 (15 months age) mice with a verified aging phenotype were obtained (Figure S3A–D, Supporting Information). Immunohistochemical staining with type 1 collagen (COL1), which indicates bone replenishment by osteoblasts on the bone surface, clearly decreased in old mice (Figure S3E, Supporting Information). By conducting treble immunofluorescence staining, the age‐dependent accumulation of apoOBs with high CD47 expression (Figure 1E) was confirmed. By screening the public data of young people and patients with osteoporosis, CD47 mRNA expression was increased in osteoporotic human osteoblasts (Figure S2E, Supporting Information), positively correlated with multiple proinflammatory (CD80 and TRAF1) and senescence biomarkers (DNMT3A), and negatively associated with the stemness marker (CARM1) in patients with osteoporosis (Figure S2F, Supporting Information). Taken together, these findings indicated that CD47 may be critically involved in apoOBs accumulation to evade osteoimmune clearance.

### 2.2 CD47 Blockade Promotes apoOBs Clearance and Reduces Age‐Related Bone Loss

**Figure 2:**
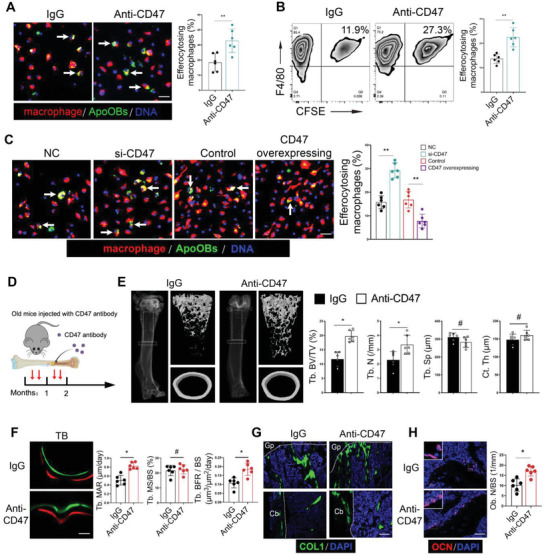
CD47 blockade strengthens apoOBs clearance and reduces age‐related bone loss. A) CFSE labeled apoOBs were cocultured with macrophages and then treated with blocking control IgG or anti‐CD47 antibodies. Macrophages were indicated by immunofluorescence staining using F4/80 antibodies. Scale bars = 100 µm. The right panel showed the quantitative percentage of efferocytosed apoOBs (*n* = 6). B) Flow cytometric analysis of efferocytosis following blockade with control IgG or anti‐CD47 antibodies with quantitative analysis at the right panel (*n* = 6). C) The knockdown of CD47 increased apoOBs clearance, whereas overexpression of CD47 decreased the efferocytosis. The right panel showed the quantitative data (*n* = 6). Scale bars = 100 µm. D) Experimental design for treating age‐related bone loss using anti‐CD47 antibodies. E) Representative micro‐CT images of the trabecular and cortical bone as well as the quantitative measurements of trabecular bone volume/tissue volume (Tb.BV/TV), trabecular number (Tb.N.), trabecular separation (Tb.Sp.), and cortical thickness (Ct.Th) in old mice treated with IgG or anti‐CD47 antibodies. F) Representative images of dynamic histomorphometry of trabecular bone (Tb) with the quantification of mineralization apposition rate (MAR), mineralizing surface/bone surface (MS/BS) and bone formation rate per unit of bone surface (BFR/BS). Scale bars = 20 µm. G) Immunofluorescence assay of COL1^+^ area on trabecular and cortical bone surface. Gp, growth plate. Cb, cortical bone. Scale bar = 100 µm. H) Representative OCN staining images and quantitative for Ob.N/BS analysis. Scale bars = 50 µm. The results are presented as the mean ± S.D. *p < 0.05; **p < 0.01; #*p* > 0.05 by one‐way ANOVA followed with Tukey multiple comparisons tests or unpaired 2‐tailed Student's *t*‐tests.

Given that CD47 is an immune checkpoint for macrophage engulfment, we next aimed to test whether CD47 blockade could promote the efferocytosis of apoOBs by macrophages and attenuate age‐related bone loss. The anti‐CD47 or anti‐SIRPα antibodies were first utilized to block the CD47‐SIRPα axis. Augmented efferocytosis was observed in apoOBs with CD47 blockade, unlike those in the control IgG (**Figure** **2**A,B). Similar results were observed in anti‐SIRPα‐pretreated macrophages (Figure S4A,B, Supporting Information). Next, both loss‐ and gain‐of‐function assays were used to determine the effects of CD47 on apoOBs clearance in vitro. CD47 knockdown cells transfected with CD47‐siRNA markedly increased the efficiency of efferocytosis, while ectopic overexpression of CD47 by the cDNA plasmid significantly impaired these effects (Figure 2C; and Figure S4C, Supporting Information). Moreover, 15‐months‐old mice were intrafemorally injected with anti‐CD47 antibodies or IgG twice a month for 2 months (Figure 2D). Although no significant difference in cortical bone thickness was detected, trabecular bone mass was pronouncedly rejuvenated as evidenced by increased bone formation, accelerated bone replenishment, and an increased number of osteoblasts on trabecular bone surfaces in animals with CD47 blockade compared to those with IgG treatment (Figure 2E–H; and Figure S4D,E, Supporting Information). In summary, our data suggest that enhancing efferocytosis by CD47 blockade or regulation might represent a viable way to prevent apoOBs from escaping osteoimmune surveillance.

### 2.3 Control of CD47 Expression by SIRT6‐Mediated Transcriptional Pausing

**Figure 3:**
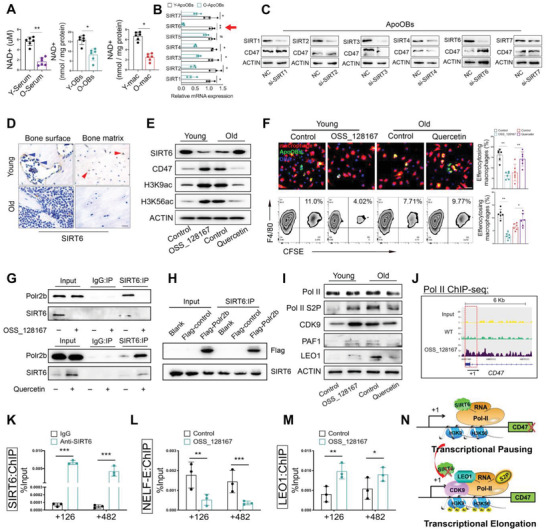
SIRT6‐mediated transcriptional pausing regulates CD47 expression. A) NAD^+^ was measured in serum, osteoblasts and macrophages from young and old mice (*n* = 6). Y, young mice. O, old mice. B) Quantitative RT‐qPCR analysis of the mRNA levels of sirtuins in young and old apoOBs (*n* = 3). C) OBs were transfected with siRNAs for SIRT1, 2, 3, 4, 6, 7. They were then induced into apoOBs for Western blot analysis. D) Representative immunostaining staining of SIRT6 in bone surface (blue arrow) and matrix (red arrow) from young and old mice. Scale bar = 50 µm. E) Western blot revealing the protein expression of SIRT6, CD47, H3K9ac, and H3K56ac with aging or SIRT6 pharmacological intervention. F) Phagocytosis assay for apoOBs efferocytosis with SIRT6 alternation (*n* = 6). Scale bar = 100 µm. G) Western blot showing co‐immunoprecipitation between SIRT6 and Polr2b with downregulation or upregulation of SIRT6. H) Coimmunoprecipitation of SIRT6 with ectopically expressed FLAG‐tagged Polr2b in 293T cells. I) Western blot showing Pol II S2P, CDK9, PAF1, and LEO1 proteins in apoOBs with aging or SIRT6 pharmacological interventions. J) ChIP‐seq assay using RNA Pol II antibodies in apoOBs showing elevated occupation of Pol II on CD47 promoter‐proximal sites. K) ChIP‐qPCR for SIRT6 on CD47 promoter‐proximal sites (*n* = 3). L) ChIP‐qPCR for NELF‐E on CD47 promoter‐proximal sites in apoOBs treated with PBS or OSS_128167 (n = 3). M) ChIP‐qPCR for LEO1 on CD47 promoter‐proximal sites in apoOBs treated with PBS or OSS_128167 (*n* = 3). N) Schematic showing the mechanism that SIRT6 inhibits CD47 transcription initiation by modulating transcriptional pausing and elongation. Results are presented as the mean ± S.D. *p < 0.05; **p < 0.01; ***p < 0.001; #*p* > 0.05 by one‐way ANOVA followed with Tukey multiple comparisons tests or unpaired 2‐tailed Student's *t*‐test.

To avoid the possible side‐effects of CD47 blockade‐induced hematotoxicity, its upstream regulatory mechanism was investigated to find an alternative approach to block this checkpoint. Since declined NAD^+^ metabolism is one of the characteristics of aging associated with many skeletal disease, NAD^+^ levels in the serum, OBs and macrophages from aged mice were detected, all of which showed decreased levels compared to those in their young littermates (**Figure** **3**A).^[^ ^24^, ^38^ ^]^ As sirtuins are NAD^+^‐dependent protein deacylases that are strongly linked to age‐related bone loss, we investigated whether sirtuins are involved in the regulation of CD47 expression and which sirtuins most clearly targets this process.^[^ ^26^ ^]^ All sirtuin genes in apoOBs and OBs during aging were simultaneously profiled. Our findings reveal that most of these genes, except for SIRT5, were remarkably decreased (Figure 3B; and Figure S5A, Supporting Information), which was also confirmed by protein detection (Figure S5B, Supporting Information). SIRT6 knockdown upregulated CD47 expression in apoOBs (Figure 3C; and Figure S5C, Supporting Information). Additionally, the decreased numbers of SIRT6^+^ and osterix^+^ (downstream targets of SIRT6) cells with aging indicated that SIRT6 deficiency might result in decreased osteoblast numbers on the bone surface (Figure 3D; and Figure S5D,E, Supporting Information). To evaluate the effect of SIRT6 on apoOBs clearance in vitro, a SIRT6‐specific inhibitor (100 µm OSS_128167) and activator (500 µm quercetin) were used.^[^ ^39^, ^40^ ^]^ As expected, OSS_128167 potently inhibited expression of SIRT6, and substantially enhanced CD47 expression as accompanied with increased acetylation of H3K9 and H3K56. On the contrary, quercetin exposure had the opposite effect (Figure 3E; and Figure S5F, Supporting Information). Notably, the efferocytosis of young apoOBs was largely abrogated in response to OSS_128167, whereas augmented efferocytosis was observed in aged apoOBs treated with quercetin as compared with the controls (Figure 3F).

To further elucidate the molecular mechanism of SIRT6‐mediated CD47 expression in apoOBs, nano‐LC‐MS/MS assays were performed to identify the interacting partners of SIRT6. Interestingly, Polr2b, a large subunits of RNA polymerase II (Pol II), was identified as a SIRT6 binding partner (Figure S5G and Table S3, Supporting Information). MYH9 was excluded because of its central role of regulating cytoskeleton reorganization in the cytoplasm, which may not directly affect the transcription of target genes. A coimmunoprecipitated (co‐IP) assay was performed in the presence or absence of OSS_128167 or quercetin to confirm the existence of binding between endogenous SIRT6 and Polr2b proteins in apoOBs (Figure 3G). Consistently, when FLAG‐tagged Polr2b was introduced in HEK293T cells, this ectopic protein was efficiently precipitated using SIRT6 antibodies (Figure 3H). Pol II pausing at promoter‐proximal regions is a widespread regulatory mechanism that occurs before proceeding toward productive elongation.^[^ ^41^ ^]^ Recent work has unveiled that Pol II pausing or release during transcription as a SIRT6‐dependent process.^[^ ^42^ ^]^ To investigate whether CD47 transcription was affected by SIRT6‐dependent transcriptional elongation via Pol II pausing, both Pol II and SIRT6 ChIP‐seq datasets were analyzed in wild‐type (WT) and SIRT6 knockout (KO) mouse embryonic stem cells. A prominent overlap between SIRT6 and Pol II was observed at the promoter‐proximal sites of CD47 (Figure S5H, Supporting Information), as well as increased Pol II binding upon SIRT6 removal (Figure S5I, Supporting Information), suggesting that SIRT6 and Pol II might cooperatively regulate CD47 transcription at its pausing site. In addition, the ChIP‐seq data on H3K9ac and H3K56ac (the SIRT6 substrates that confer its pausing function), as well as on LEO1 (a transcription elongation factor) showed large enrichment at the promoter‐proximal regions of CD47 in SIRT6‐deleted cells (Figure S5J,K, Supporting Information). To validate whether SIRT6 could modulate these relevant functional proteins in apoOBs, transcription elongation factors (Pol II S2P, CDK9, PAF1, LEO1) (Figure 3I; and Figure S5L, Supporting Information) and transcriptional pausing factors (NELF‐E) in the chromatin fraction (Figure S5M, Supporting Information) in response to different SIRT6 expression were detected, which in turn suggests the regulatory role of SIRT6‐dependent transcription pausing in apoOBs. To ascertain that the SIRT6‐modutory transcription pausing mechanism is indeed responsible for CD47 expression, a ChIP‐seq assay using antibodies targeting RNA Pol II in the absence or presence of OSS_128167 treated OBs was performed, which revealed increased enrichment of Pol II binding to the CD47 promoter‐proximal region upon SIRT6 downregulation (Figure 3J; and Table S4, Supporting Information). To further confirm this, ChIP‐qPCR was performed in OBs and a dual‐luciferase assay in HEK293T cells, both of which validated SIRT6 binding at the promoter‐proximal regions of CD47 (Figure 3K; and Figure S5N, Supporting Information). Moreover, NELF‐E and LEO1 ChIP‐qPCR in SIRT6‐deficient OBs also revealed a decrease or strong enrichment of the CD47 promoter, respectively (Figure 3L,M). Hence, our data showed that SIRT6 retarded CD47 expression by decreasing promoter‐proximal acetylation of H3K9 and H3K56, as well as maintaining their transcriptional pausing (Figure 3N).

Having documented the roles of SIRT6 in suppressing CD47 in apoOBs, we next set out to explore the roles of SIRT6 affecting SIRPα expression in macrophages and efferocytosis. Aged macrophages displayed proinflammatory macrophage polarization and defective efferocytosis (Figure S6A–C, Supporting Information). Pharmacological intervention of SIRT6 in macrophages revealed the opposite expression patterns between SIRT6 and SIRPα as well as H3K9 and H3K56 acetylation (Figure S6D, Supporting Information). Young macrophages pretreated with OSS_128167 displayed impaired engulfment efficiency, whereas quercetin restored the impaired efferocytosis of aged macrophages (Figure S6E, Supporting Information). Public ChIP‐seq analysis displayed that both Pol II and SIRT6 were observed near the proximal‐promoter of SIRPα, as well as by H3K9ac and H3K56ac, LEO1 ChIP‐seq analysis (Figure S6F, Supporting Information). SIRT6, NELF‐E, or LEO1 ChIP‐qPCR assays were performed on macrophages (Figure S6G–I, Supporting Information), which further confirmed the regulation of SIRPα in SIRT6‐dependent transcriptional pausing. Together, these results reveal that SIRT6 influences the efferocytosis of macrophages by regulating SIRPα transcriptional pausing.

### 2.4 Increased apoOBs Accumulation and Bone Loss by Deleting SIRT6 in Osteoblasts

**Figure 4:**
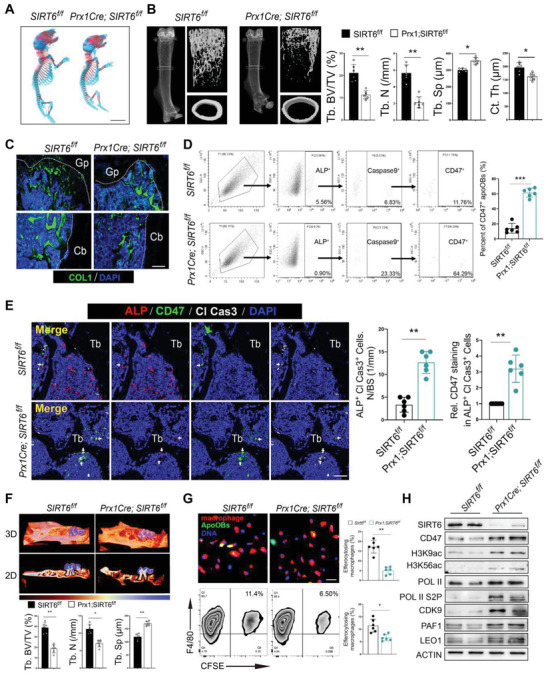
SIRT6 deletion in osteoblasts using Prx1cre leads to exacerbated bone loss and impaired apoOBs clearance. A) Alizarin red and Alcian blue staining displaying the whole‐mount skeletal of newborn *Prx1cre;SIRT6^f/f^* and *SIRT6^f/f^* mice. Scale bar = 5 mm. B) Representative micro‐CT images of the trabecular and cortical bone and quantitative measurements of Tb.BV/TV, Tb.N, Tb.Sp, and Ct.Th in *Prx1cre;SIRT6^f/f^* and *SIRT6^f/f^* mice. *n* = 6. C) Immunostaining of COL1^+^ area on trabecular and cortical bone surface (*n* = 6). Scale bar = 100 µm. D) The gating strategy to isolate and compare the difference of ALP^+^ Caspase9^+^ CD47^+^cells in bone marrow from *Prx1cre;SIRT6^f/f^* and *SIRT6^f/f^* mice. The right panel showed the quantitative data (*n* = 6). E) Representative images of ALP, cleaved caspase3 and CD47 immunostaining on bone surface in *Prx1cre;SIRT6^f/f^* and *SIRT6^f/f^* mice. Right panel: quantitative measurements of apoOBs numbers and CD47^+^ apoOBs staining on bone surface (*n* = 6). Scale bar = 25 µm. F) Representative micro‐CT images of new bone fill in first maxillary molar tooth extraction socket after 2 weeks by color‐coded thickness maps. The color changes from red to blue denote a gradual elevation in bone thickness. Quantitative measurements of Tb.BV/TV, Tb.N, Tb.Sp for new bone in extraction socket from *Prx1cre;SIRT6^f/f^* and *SIRT6^f/f^* mice. *n* = 6. G) OBs were collected from *SIRT6^f/f^* and *Prx1cre;SIRT6^f/f^* and induced into apoOBs for phagocytosis assay (*n* = 6). Scale bar = 100 µm. H) Proteins of SIRT6, CD47, H3K9ac, H3K56ac, Pol II, Pol II S2P, CDK9, PAF1, and LEO1 in apoOBs from *Prx1cre;SIRT6^f/f^* and *SIRT6^f/f^* mice. Results are presented as the mean ± S.D. *p < 0.05; **p < 0.01; ***p < 0.001; #*p* > 0.05 by unpaired 2‐tailed Student's *t*‐test.

To elucidate the in vivo effects of SIRT6 on apoOBs clearance and bone homeostasis, conditional SIRT6 knockout mice were generated from skeletal progenitors using *Prx1cre* mice. Skeletal staining at P1 littermates indicated that *Prx1cre;SIRT6^f/f^* mice were underdeveloped and showed smaller skeletons than *SIRT6^f/f^* mice (**Figure** **4**A). Micro‐CT and fluorescence labeling analyses revealed significant bone loss and reduced bone formation in the trabecular and endosteal bone in *Prx1cre;SIRT6^f/f^* mice compared to *SIRT6^f/f^* controls (Figure 4B; and Figure S7A, Supporting Information). Furthermore, *Prx1cre;SIRT6^f/f^* mice exhibited decreased COL1^+^ bone area, trabecular bone mass, and osteoblast numbers (Figure 4C; and Figure S7B,C, Supporting Information). Notably, flow cytometry assay revealed an increased ratio of CD47 positive apoOBs in *Prx1cre;SIRT6^f/f^* mice (Figure 4D). Increased cleaved caspase3^+^ apoOBs on the bone surface and higher amounts of CD47^+^ osteoblasts were occurred in *Prx1cre;SIRT6^f/f^* mice (Figure 4E), thus suggesting that CD47‐involved apoOBs clearance was affected by SIRT6 deficiency. To determine whether osteoblastic SIRT6 deletion affects bone regeneration, a bone defect model was constructed by removing the first molar of the maxilla. As shown in Figure 4F, new bone formation in the tooth socket was remarkably compromised after tooth extraction in *Prx1cre;SIRT6^f/f^* mice, which was supported by reduced woven bone formation (Figure S7D, Supporting Information). To further determine the in vitro effect of SIRT6 deficiency on efferocytosis, apoOBs from *SIRT6^f/f^* and *Prx1cre;SIRT6^f/f^* mice were cultured for phagocytosis assays, which displayed impaired efferocytosis due to the absence of SIRT6 (Figure 4G). Consistent with the above data (Figure 3E), SIRT6 deficiency led to upregulated CD47 expression, increased H3K9/H3K56 acetylation, and enhanced transcriptional elongation (Figure 4H; and Figure S7E, Supporting Information). Collectively, these data indicate that osteoblastic SIRT6 deficiency contributes to accelerated bone loss and adverse bone regeneration, possibly due to increased CD47 expression in osteoblasts and impaired clearance of apoOBs.

### 2.5 ApoOBs with SIRT6 Deficiency Attenuate Macrophage Recruitment

**Figure 5:**
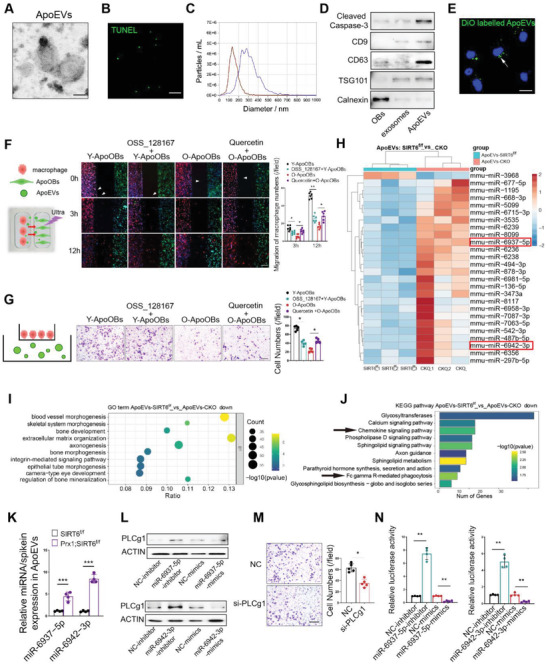
ApoOBs deliver miRNAs‐loaded apoVs to regulate macrophage recruitment. A) Representative image of the transmission electron microscope (TEM) showing apoVs morphology. Scale bar, 100 nm. B) Representative microscopy images of apoVs by TUNEL staining. Scale bar = 20 µm. C) Nanoparticle tracking analysis (NTA) displaying the different size distribution between apoVs and exosomes. Red, exosomes. Purple, apoVs. D) Western blot analysis of osteoblasts, exosomes from osteoblasts, and apoVs from apoOBs. Cleaved caspase 3 is an apoptosis marker; CD9, CD63, and TSG101 are exosome markers; Calnexin is a cytosolic marker. E) DiO‐labeled apoVs (indicated by the white arrow) incorporated into macrophages were visualized by fluorescent microscopy analysis. Scale bar = 20 µm. F) Representative immunofluorescent staining images of macrophages (DiI, Red) toward apoOBs (CFSE, Green) under culture‐insert 2 well system. Arrowheads indicates apoVs. Scale bar = 200 µm. G) The numbers of infiltrated macrophages from upper chamber with the induction of apoVs in lower chamber. Scale bar = 200 µm. H) Hierarchical clustering heatmap of different miRNAs expression in apoVs (*p* value<0.05, FDR<0.05) from *SIRT6^f/f^* and *Prx1cre;SIRT6^f/f^* apoOBs. The columns represent individual replicates. Red box, two miRNAs involved in the regulation of the chemokine signaling pathway. I) Gene ontology (GO) analysis for targeted gene by upregulated miRNAs. The bubble chart indicates the top ten enriched terms. J) The Kyoto Encyclopedia of Genes and Genomes (KEGG) analysis was presented as a bar chart. K) The miR‐6937‐5p and miR‐6942‐3p loaded in apoVs from *SIRT6^f/f^* and *Prx1cre;SIRT6^f/f^* mice were examined by RT‐qPCR. L) Western blot showing the PLCg1 expression in miR‐6937‐5p and miR‐6942‐53p inhibitor or mimics treated macrophages. M) The alternation of macrophage infiltration with si‐RNA PLCg1 interference. Scale bar = 200 µm. N) Construction of PLCg1 plasmid vectors for dual‐luciferase reporter assays. Results are presented as the mean ± S.D. *p < 0.05; **p < 0.01; ***p < 0.001; #*p* > 0.05 by one‐way ANOVA followed with Tukey multiple comparisons tests or unpaired 2‐tailed Student's *t*‐tests.

By scrutinizing the spatial distribution of apoptotic cells and macrophages on bone surface in vivo, macrophages distributed around the bone surface were found to be lower in numbers in aged mice and *Prx1cre;SIRT6^f/f^* mice as compared to their counterparts (Figure S8A,B, Supporting Information). This interesting phenomenon prompted us to investigate whether impaired efferocytosis was associated with reduced macrophages recruitment, which might be relevant to the intercellular crosstalk between apoOBs and macrophages. Considering extracellular vesicles are the main components of intercellular communication, we hypothesized that apoOBs might regulate macrophages recruitment by releasing EVs, which are defined as apoptotic osteoblasts‐derived vesicles (apoVs). To decipher this physiological process, apoVs were isolated from apoOBs by ultracentrifugation and phenotypically confirmed by transmission electron microscopy (TEM) (**Figure** **5**A), positive TUNEL staining (Figure 5B), and size distribution (Figure 5C).^[^ ^43^ ^]^ Moreover, these apoVs were characterized by apoptosis‐ and EVs‐associated markers (Figure 5D) and were efficiently captured by macrophages (Figure 5E). To unveil whether SIRT6 intervention in apoOBs influences macrophage recruitment, a culture‐insert 2‐well system was used, revealing that migrated macrophages were markedly decreased in OSS_128167‐treated apoOBs from young mice, whereas quercetin treatment enhanced macrophage recruitment toward aged apoOBs (Figure 5F). Similar findings were independently verified using the transwell system (Figure 5G). Since the miRNAs composition of EVs is vital in cell communication and bone remodeling, apoVs from *SIRT6^f/f^* and *Prx1cre;SIRT6^f/f^* were prepared for miRNA‐seq to identify functional miRNA candidates. A total of 1377 miRNAs were identified (Table S5, Supporting Information), with differential miRNAs identified using Volcano plot (Figure S8C, Supporting Information). As shown in Figure 5H (*p* value<0.05, FDR<0.05), most miRNAs in apoVs from mutant animal were upregulated. Notably, the putative targets of these upregulated miRNAs were significantly enriched during bone and vessel morphogenesis and development (Figure 5I; and Table S6, Supporting Information). Further pathway analysis revealed that chemokine signaling pathway and Fc gamma R‐mediated phagocytosis associated with macrophages engulfment were markedly affected in response to SIRT6 deletion (Figure 5J; and Table S7, Supporting Information). Based on the signature of chemokine signaling pathway, miR‐6937‐5p and miR‐6942‐53p were screened among these differentially expressed miRNAs that were mainly involved in the regulation of the chemokine pathway. The enrichment of miR‐6937‐5p and miR‐6942‐3p in apoOBs and apoVs from *SIRT6^f/f^* and *Prx1cre;SIRT6^f/f^* mice was independently verified by qRT‐PCR assay (Figure 5K; and Figure S8D, Supporting Information). Moreover, using Miranda and TargetScan, PLCg1 was predicted to be a shared target gene of miR‐6937‐5p and miR‐6942‐53p which are key functional modulators in adaptive immune responses, bone homeostasis, and chemotaxis.^[^ ^44^ ^]^ To illustrate the role of PLCg1 in this process, apoVs were cocultured with macrophages, which showed a drastic decrease in PLCg1 protein in the *Prx1cre;SIRT6^f/f^* apoVs‐treated group (Figure S8E, Supporting Information). Additionally, by introducing either miR‐6942‐3p inhibitors or mimics into macrophages, significantly increased or decreased PLCg1 expressions were observed, respectively. Similar changes were observed in miR‐6937‐5p inhibitors‐ or mimics‐treated macrophages (Figure 5L; and Figure S8F, Supporting Information). Moreover, PLCg1 knockdown potently suppressed macrophages migration in vitro, suggesting a positive effect of PLCg1 on macrophage chemotaxis (Figure 5M). Importantly, to validate the direct binding sites between miR‐6937‐5p or miR‐6942‐3p and PLCg1, pGL3‐basic luciferase reporter plasmid vectors were constructed. We found that the ectopic overexpression of miR‐6937‐5p or miR‐6942‐3p substantially reduced luciferase activities, and vice versa (Figure 5N). Overall, these results indicate that apoOBs without SIRT6 impair macrophage recruitment by releasing miRNAs‐loaded apoVs, which in turn facilitates the evasion of osteoimmune clearance by apoOBs.

### 2.6 Impaired Efferocytosis by SIRT6‐Deleted Macrophages Leads to Profound Bone Loss

**Figure 6:**
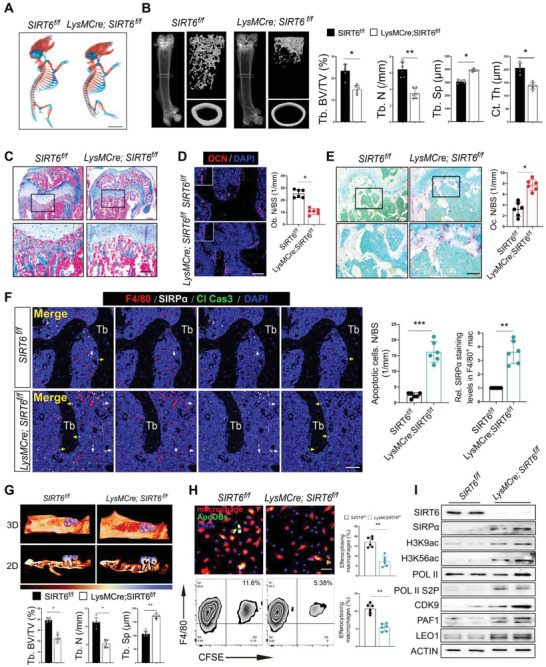
Loss of SIRT6 in macrophages inhibits efferocytosis and reduces bone mass. A) Alizarin red and Alcian blue staining displaying the whole‐mount skeletal of *LysMcre;SIRT6^f/f^* and *SIRT6^f/f^* mice at P1. Scale bar = 5 mm. B) The micro‐CT images of trabecular and cortical bone of distal femurs from *LysMcre;SIRT6^f/f^* and *SIRT6^f/f^* mice for Tb.BV/TV, Tb.N, Tb.Sp, and Ct.Th. *n* = 6. C) Representative masson trichrome staining showing the trabecular bone difference in *LysMcre;SIRT6^f/f^* and *SIRT6^f/f^* mice. Scale bars = 200 µm. D) Representative OCN staining and quantitative for Ob.N/BS analysis (*n* = 6). Scale bars = 50 µm. E) TRAP staining of the femurs from *LysMcre;SIRT6^f/f^* and *SIRT6^f/f^* mice and quantification of osteoclast surface per bone surface (*n* = 6). Scale bars = 100 µm. F) Representative immunostaining image of F4/80^+^, SIRPα^+^, and cleaved caspase3^+^ cells in bone marrow from *LysMcre;SIRT6^f/f^* and *SIRT6^f/f^* mice. The right panel shows the quantitative measurements of apoptotic cell numbers on bone surface and SIRPα^+^ macrophages (*n* = 6). Yellow arrow, apoptotic cells. White arrow, SIRPα^+^ macrophages. Scale bar = 25 µm. G) Representative 2D and 3D reconstruction of extraction socket in *LysMcre;SIRT6^f/f^* and *SIRT6^f/f^* mice by color coded thickness maps and quantitative measurements of Tb.BV/TV, Tb.N, Tb.Sp for new bone fill. *n* = 6. H) Macrophages were obtained from *SIRT6^f/f^* and *LysMcre;SIRT6^f/f^* mice and cocultured with apoOBs for phagocytosis assay (*n* = 6). Scale bar = 100 µm. I) Proteins of SIRT6, SIRPα, H3K9ac, H3K56ac, Pol II, Pol II S2P, CDK9, PAF1, and LEO1 by macrophages from *LysMcre;SIRT6^f/f^* and *SIRT6^f/f^* mice. Results are presented as the mean ± S.D. *p < 0.05; **p < 0.01; ***p < 0.001; #*p* > 0.05 by unpaired 2‐tailed Student's *t*‐test.

To further explore the influence of SIRT6 in macrophages on apoOBs clearance in vivo, myeloid‐specific SIRT6 knockout mice (*LysMcre;SIRT6^f/f^* mice) were generated. No obvious differences in skeletal development were observed between SIRT6^f/f^ and *LysMcre;SIRT6^f/f^* mice (**Figure** **6**A). However, micro‐CT and dynamic histomorphometric analysis revealed dramatically decreased bone mass and declined bone formation in the trabecular and endosteal bone of *LysMcre;SIRT6^f/f^* mice (Figure 6B; and Figure S9A, Supporting Information). Masson's trichrome and immunofluorescence staining showed that both trabecular bone mass and osteoblasts were markedly reduced in *LysMcre;SIRT6^f/f^* mice (Figure 6C,D). Since osteoclasts differentiate from myeloid‐derived macrophages, osteoclastic activity was also examined using TRAP staining, which showed significantly enhanced osteoclastic activity in *LysMcre;SIRT6^f/f^* mice (Figure 6E). By performing treble immunofluorescence staining (Figure 6F), much higher numbers of apoptotic cells on the bone surface and SIRPα^+^ macrophages were observed in *LysMcre;SIRT6^f/f^* mice, suggesting a dysfunctional clearance of apoptotic cells might be associated with highly expressed SIRPα in macrophages due to SIRT6 deletion. To determine the effect of macrophage‐specific SIRT6 deletion on bone regeneration, *LysMcre;SIRT6^f/f^* mice receiving tooth extraction surgery displayed substantially less woven bone but increased osteoclasts than those in *SIRT6^f/f^* mice (Figure 6G; and Figure S9B,C, Supporting Information). In line with these in vivo findings, SIRT6‐deleted macrophages from *LysMcre;SIRT6^f/f^* mice failed to efficiently efferocytose apoOBs in vitro (Figure 6H). Moreover, SIRT6 deficiency resulted in increased SIRPα proteins and histone acetylation and transcriptional elongation activity in macrophages (Figure 6I; and Figure S9D, Supporting Information). Collectively, our data reveal that SIRT6‐deleted macrophages weaken their efferocytosis ability due to enhancement of SIRPα, thus resulting in upregulated bone loss and downregulated bone regeneration.

### 2.7 Cell‐Targeting Delivery of SIRT6 Robustly Increases Bone Mass

**Figure 7:**
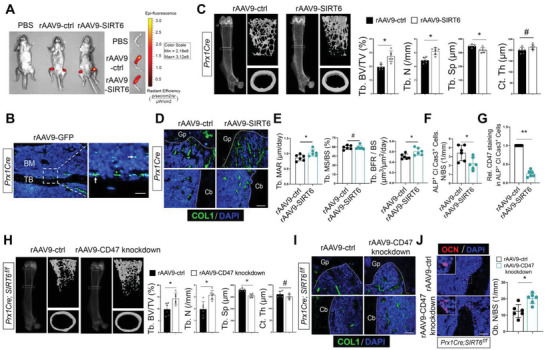
OBs‐targeting SIRT6 overexpression increases bone mass in *Prx1cre* mice. A) rAAV9‐SIRT6 and rAAV9‐control vectors were intrafemorally injected into bone marrow cavity of 3‐month‐old *Prx1cre* mice that were monitored by IVIS optical imaging. The *Y*‐axis indicates radiant efficiency (p/s/cm^2^/sr/µW/cm^2^). B) EGFP expression was assessed by fluorescence microscopy. Scale bars = 50 µm. White arrow, EGFP^+^ osteoblasts. C) Femoral trabecular bone mass and cortical bone thickness in rAAV9‐control‐ and rAAV9‐SIRT6‐treated *Prx1cre* mice were assessed by 3D reconstruction and relative quantification of micro‐CT (*n* = 6). D) Immunofluorescence assay of COL1^+^ area reflecting the state of new bone replenishment in rAAV9‐control‐ and rAAV9‐SIRT6‐treated *Prx1cre* mice. Scale bars: 100 µm. E) Quantification of Tb with MAR, MS/BS, and BFR/BS were displayed. F) Quantification of apoOBs numbers on bone surface. *n* = 6. G) Quantitative measurements of CD47 in apoOBs (*n* = 6). H) Representative micro‐CT images of trabecular and cortical bone and quantitative measurements of Tb.BV/TV, Tb.N, Tb.Sp, and Ct.Th in rAAV9‐control‐ and rAAV9‐CD47 knockdown‐treated *Prx1cre;SIRT6^f/f^* mice (*n* = 6). I) Immunofluorescence assay of COL1^+^ area on trabecular and cortical bone surface (*n* = 6). Gp, growth plate. Cb, cortical bone. Scale bar = 100 µm. J) Representative immunofluorescence staining images of OCN and quantitative for Ob.N/BS analysis (*n* = 6). Scale bars = 50 µm. Results are presented as the mean ± S.D. *p < 0.05; **p < 0.01; #*p* > 0.05 by unpaired 2‐tailed Student's *t*‐test.

To avoid the accumulation of apoOBs in the context of age‐related bone loss, therapeutic interventions that target SIRT6 to monitor the CD47‐SIRPα checkpoint might be potent in a cell‐specific or tissue‐specific strategy to restore bone homeostasis. To confirm the cell‐specific role of SIRT6, Cre‐dependent recombinant rAAV9‐mediated gene transduction vectors were designed and injected into *Prx1cre* or *LysMcre* mice. In this case, rAAV9‐transduced SIRT6 vectors can recognize Cre^+^ cells and enhance intracellular SIRT6 expression. Accordingly, *Prx1cre* mice at 6 months old were intrafemorally injected with rAAV9‐mediated SIRT6 overexpression (rAAV9‐SIRT6‐EGFP) or rAAV9‐mediated EGFP expression (control). Body weight and blood cells, including white blood cells, red blood cells, and platelet, were assessed, which revealed no significant differences between the control and SIRT6‐manipulated mice (Figure S10A,B, Supporting Information). The fluorescence of EGFP was monitored by IVIS optical imaging in the entire body (**Figure** **7**A) and its protein in the femur was detected on the bone surface using fluorescence microscopy (Figure 7B). After validating the efficiency of SIRT6 expression by Western blot (Figure S10C, Supporting Information), the mice treated with rAAV9‐SIRT6 showed a marked increase in trabecular bone mass, but no significant discrepancy in cortical bone thickness compared to rAAV9‐control‐treated *Prx1cre* mice (Figure 7C; and Figure S10D, Supporting Information). Likewise, the activity of osteogenesis was enhanced as evidenced by COL1^+^ bone area, increased bone formation and osteoblasts on bone surface in rAAV9‐SIRT6‐treated *Prx1cre* mice (Figure 7D,E; and Figure S10E–G, Supporting Information). We also examined the effect of rAAV9‐SIRT6 on osteoclastic differentiation and found no significant difference (Figure S10H, Supporting Information). Importantly, compared to the control counterparts, rAAV9‐SIRT6 treated mice showed fewer apoOBs and lower CD47^+^ apoOBs residing on the bone surface (Figure 7F,G). To further ascertain the effects of SIRT6 on bone homeostasis by targeting CD47, we repressed CD47 expression in *Prx1cre;SIRT6^f/f^* mice by transducing another cre‐dependent rAAV9‐CD47 knockdown vectors. After verifying the fluorescence of EGFP by IVIS optical imaging (Figure S10I, Supporting Information) and knockdown efficiency of CD47 by Western blot (Figure S10J, Supporting Information), micro‐CT analysis indicated that as expected, the originally low bone mass in *Prx1cre;SIRT6^f/f^* mice was relatively increased once injected with rAAV9‐CD47 knockdown (Figure 7H). Analogous to the increased bone formation (Figure S10K, Supporting Information), the COL1^+^ bone area and osteoblasts on bone surface were also alleviated in rAAV9‐CD47 knockdown‐treated animals (Figure 7I,J). Together, our results reveal a causal role of SIRT6 in retarding CD47 expression and promoting apoOBs clearance to restore normal bone homeostasis.

Next, we investigated whether targeted delivery of SIRT6 into macrophages might also enhance efferocytosis and increase bone mass in vivo. The *LysMcre* mice were injected with rAAV9‐SIRT6 to enhance SIRT6 in macrophages. After examining body wight and blood cells (Figure S11A,B, Supporting Information), macrophage‐specific overexpression of STRT6 in vivo was validated in macrophages extracted from animal receiving rAAV9‐SIRT6 vectors delivery (Figure S11C,D, Supporting Information). Micro‐CT analysis revealed increased bone mass in rAAV9‐SIRT6‐treated *LysMcre* mice (Figure S11E, Supporting Information). Moreover, accelerated bone formation in the trabecular and endosteal bone, elevated COL1^+^ trabecular bone and increased osteoblasts were detected in femurs transduced with rAAV9‐SIRT6 along with decreased osteoclastic differentiation (Figure S11F–J, Supporting Information). Importantly, as expected, *LysMcre* mice with rAAV9‐SIRT6 delivery showed declined apoOBs on the bone surface and downregulated SIRPα^+^ macrophages in bone marrow (Figure S11K,L, Supporting Information). Collectively, these results provide strong evidence that the mechanism of SIRT6 regulated CD47‐SIRPα checkpoint is a critically physical process to clear apoOBs and suggest that delivery of SIRT6 in osteoblasts or macrophages holds therapeutic potential to rejuvenate bone homeostasis.

### 2.8 Delivering SIRT6 Activator Prevents Age‐Related Bone Loss

**Figure 8:**
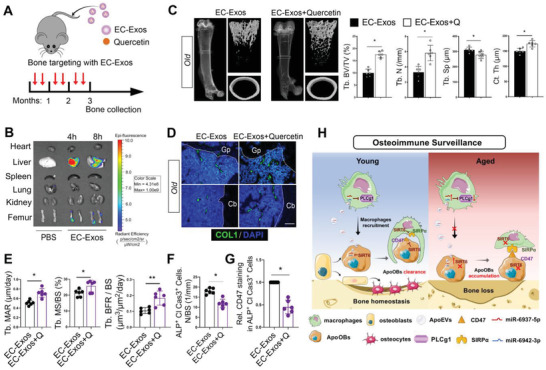
EC‐Exos delivery systems with a SIRT6‐dependent drug loading. A) Schematic illustration of quercetin loaded EC‐Exos and injection frequency. B) Biophotonic images of fluorescence signal in the heart, liver, spleen, lung, kidney, and femur after injection of PBS and DiI‐labeled EC‐Exos. The *Y*‐axis indicates radiant efficiency (p/s/cm^2^/sr/µW/cm^2^). C) Representative micro‐CT images and quantitative analysis of the distal femur in old mice treated with EC‐Exos or quercetin loaded EC‐Exos (*n* = 6). D) Immunofluorescence assay of COL1^+^ area reflected the state of new bone replenishment in EC‐Exos or quercetin loaded EC‐Exos injected old mice. Scale bars = 100 µm. E) Quantification of Tb with MAR, MS/BS, and BFR/BS. F) apoOBs numbers and G) CD47^+^ apoOBs in EC‐Exos or quercetin‐loaded EC‐Exos injected mice (*n* = 6). H) Schematic model: osteoimmune surveillance of SIRT6‐regulatory checkpoint and macrophage migration to promote apoOBs efferocytosis and prevent aged‐related bone loss. Results are presented as the mean ± S.D. *p < 0.05; **p < 0.01 by unpaired 2‐tailed Student's *t*‐test.

Recent work has established that exosomes are excellent carriers for drug‐delivery systems; in particular, vascular endothelial cells derived exosomes (EC‐Exos) possess highly bone‐targeting efficiency.^[^ ^45^ ^]^ Having independently revealed the essential roles of SRIT6 in apoOBs or macrophages, we next wanted to determine whether delivering SIRT6 activator in bone tissue could prevent age‐related bone loss via EC‐Exos loaded drug strategy. The SIRT6 activator (quercetin) was loaded into EC‐Exos by electroporation, which was subsequently examined under TEM, showing no evidence of morphological changes (Figure S12A, Supporting Information). The 15‐months old mice were intravenously injected with quercetin loaded EC‐Exos twice a month for 3 months (**Figure** **8**A) and well tolerated with no significant weight loss and hematotoxicity (Figure S12B,C, Supporting Information). Consistent with previous study, high delivery efficiency of EC‐Exos for bone tissue was visualized using IVIS optical imaging (Figure 8B). Three months later, old mice with quercetin‐loaded EC‐Exos exhibited increased trabecular bone accrual, cortical bone thickness, bone formation, COL1^+^ bone area, and osteoblasts on the bone surface as compared to those receiving EC‐Exos, as well as reduced osteoclasts (Figure 8C–E; and Figure S12D–H, Supporting Information). Importantly, recovered efferocytosis and fewer CD47^+^ apoOBs in old mice were observed following treatment with quercetin‐loaded EC‐Exos (Figure 8F,G). In addition, a SIRT6 inhibitor (OSS_128167) was encapsulated into EC‐Exos and delivered to young mice at 3 months of age. Consequently, OSS_128167‐loaded EC‐Exos markedly accelerated bone loss and apoOBs accumulation (Figure S13A–I, Supporting Information). Collectively, our results reveal that therapeutic activation of SIRT6 in bone tissue robustly promoted apoOBs clearance and reversed age‐related bone loss.

## 3 Discussion

The accumulation of apoOBs with age leads to pro‐inflammatory conditions and progressive bone loss, whereas in young organisms, apoOBs are subjected to immediate macrophage efferocytosis to maintain the bone homeostasis.^[^ ^6^, ^8^ ^]^ The underlying mechanism that the dysregulation of efferocytosis results in apoOBs accumulation during immunosenescence remains unknown. Here, we identified that CD47 is highly expressed in aged apoOBs and help them evade osteoimmune surveillance. In the combination of genetic, sequencing, and pharmacologic approaches, we revealed that CD47‐SIRPα checkpoint was controlled by SIRT6 via the regulation of transcriptional pausing and its deficiency accounted for the pronouncedly accumulated apoOBs and age‐related bone loss. Moreover, SIRT6‐deleted apoOBs resulted in delayed macrophages migration by releasing miRNAs‐loaded apoVs (Figure 8H). Importantly, cre‐dependent rAAV9 gene delivery and EC‐Exos drug delivery systems provided precise targeting strategies to enhance SIRT6 expression for preventing age‐related bone loss. These findings reveal the importance of osteoimmune surveillance of apoOBs clearance in bone aging and provide two therapeutical strategies for delaying age‐related bone loss.

After completing the bone‐forming function, only a minority of osteoblasts become osteocytes or lining cells, whereas ≈50–70% of osteoblasts underwent apoptosis.^[^ ^46^, ^47^ ^]^ The previous identification of apoOBs in vivo was examined by histological observation including TUNEL staining and osteoblastic morphology (e.g., cuboidal shape, apical location of their nucleus, and the juxtaposition with osteoid), which provide limited information about time trajectory for apoOBs.^[^ ^46^ ^]^ Although an in vitro study demonstrated that apoptosis occurred in matured osteoblasts, they did not exclude a possible contribution of pre‐OBs shift to apoOBs. By exploiting scRNA‐seq analysis at single‐cell resolution, we mapped the osteoblast differentiation trajectory and identified a subcluster of OLCs as apoOBs that were temporally split out during the differentiation of matured OBs toward osteocytes. However, it should be pointed out that although mitochondrial genes have been previously used to identify apoptotic cells for scRNA‐seq analysis in several studies, cluster 3 of highly expressing mitochondrial genes in our study could not be fully identified as apoOBs, but probably include the damaged cells because cell damage inevitably occurs during isolation, flow cytometric procedures, and ongoing self‐destruction, which also leads to increased mitochondrial transcripts.^[^ ^35^, ^36^, ^48^ ^]^ Despite our scRNA‐seq data failing to obtain the difference of apoOBs between young and old OLCs, intracellular flow cytometry assay and treble immunofluorescence staining supported the notion of age‐dependent accumulation of apoOBs.

Apoptotic cells mount a rapid immune response to finish their clearance by releasing various inflammatory cytokines, chemokines, and nucleotides.^[^ ^49^, ^50^ ^]^ However, the activated immune cells might be incapable of executing their ability of engulfment in aged tissue, thus leading to a chronically inflammatory status. Emerging evidence has showcased the potency of CD47‐SIRPα checkpoint for mediating efferocytosis to escape innate immune surveillance through macrophage phagocytosis; moreover, either antibody blockade or interfering levels of CD47 are capable of influencing phagocytosis.^[^ ^17^, ^22^, ^51^ ^]^ The current study shows for the first time that aged apoOBs highly express CD47 and that checkpoint blockade promotes apoOBs elimination and reduces bone loss. Nevertheless, we used SIRT6, but not CD47, as our following therapeutic target because the potential risk of hematotoxicity of CD47 targeting agents still cannot be avoided despite various methods being used to mitigate such toxicity (particularly anemia).^[^ ^52^ ^]^ By exploring the upstream regulator, the longevity gene SIRT6 was screened as a monitor of the CD47 checkpoint via the regulatory mechanism of transcriptional pausing. Considering the role of SIRT6 in maintaining metabolism and lifespan, modulating SIRT6 to prevent age‐related diseases may be more promising for clinical applications than targeting CD47.

Although extracellular vesicles (EVs)‐mediated signal transduction including DNA, mRNA, proteins, and microRNAs (miRNAs) has been largely reported in recent years, the mechanisms involved in apoVs‐mediated intercellular transport between apoptotic cells and neighboring cells remain elusive.^[^ ^53^ ^]^ Based on the different sizes of vesicles, apoVs could be divided into apoptotic bodies (apoBDs, 1–5 µm in diameter), apoptotic microvesicles (apoMVs, 0.1–1 µm in diameter), and apoptotic exosomes (apoExos, < 150 nm in diameter).^[^ ^54^ ^]^ It should be noted that these subtypes of apoVs from different cell types executed different functions depending on the cargos loaded. In this study, we proposed that apoVs secreted by aged apoOBs cause a phenotypic shift of fewer macrophages toward the bone surface. The alternation of SIRT6‐mediated miRNAs transferred by apoVs is responsible for chemotaxis signaling regulation and the subsequent recruitment of macrophages. Therefore, these findings extend the current knowledge of “find‐me signals” that apoVs could be loaded with different miRNAs and trigger distinct responses of macrophage recruitment.^[^ ^55^ ^]^

To explore the in vivo role of SIRT6‐regulatory mechanism in osteoimmune surveillance, osteoblast‐, and macrophage‐specific SIRT6 knockout mice were constructed, and the loss of SIRT6 consequently led to impaired efferocytosis and marked bone loss. Intriguingly, combined with recent reports on efferocytosis‐mediated embryonic development of drosophila, we observed a defective postnatal skeleton in *Prx1cre;SIRT6^f/f^* mice, suggesting the potential role of SRIT6‐regulatory efferocytosis in skeletal development.^[^ ^56^ ^]^ Moreover, we also observed increased osteoclastic activity in *LysMcre;SIRT6^f/f^* mice as compared to the *SIRT6^f/f^* mice. This difference may be due to the increased SIRPα expression in SRIT6‐deleted macrophages, since activation of SIRPα was involved in macrophage–macrophage adhesion/fusion leading to multinucleation.^[^ ^57^ ^]^

Having determined the critical role of SIRT6 in regulating apoOBs clearance, we next sought to explore therapeutical strategies by enhancing SIRT6 expression in the context of age‐related bone loss. Considering the safety and efficacy of rAAV vectors in preclinical and clinical investigations, rAAV9‐SIRT6 was intrafemorally injected into *Prx1cre* or *LysMcre* mice. Our data support the hypothesis that SIRT6 overexpression promotes apoOBs clearance and restores bone mass.^[^ ^58^, ^59^ ^]^ Apart from serving as biological medicines, exosomes have natural specific tissue targeting ability with low toxicity and antigenicity.^[^ ^60^ ^]^ We selected more efficient bone targeting exosomes, EC‐Exos that were loaded with SIRT6‐specific activators, quercetin.^[^ ^40^, ^45^ ^]^ Interestingly, quercetin combined with dasatinib as a senolytic cocktail has now achieved unprecedented preclinical outcomes by targeting immune surveillance to remove senescent cells in aged diseases.^[^ ^61^, ^62^ ^]^ In our study, quercetin‐loaded EC‐Exos showed a great effect in bone targeting and prevention of age‐related bone loss, suggesting that this delivery strategy not only affects apoOBs clearance but, potentially, also removes senescent cells.

Nevertheless, this study has several limitations that should be addressed in future studies. First, since *LysMCre* mice expressing Cre recombinase were used to trace myeloid lineage cells, such as neutrophils, macrophages, and osteoclasts, impaired macrophage efferocytosis may not be the only reason for marked bone loss despite *LysMcre;SIRT6^f/f^* mice showing accumulated apoOBs on bone surface. Second, *Prx1cre;SIRT6^f/f^* mice exhibited developmental retardation of skeletal system, but the underlying mechanism of SIRT6‐mediated BMSCs function for bone development remains undetermined. Collectively, our findings uncover that aging enables apoOBs to evade osteoimmune surveillance accounting for the imbalanced bone homeostasis and targeting the regulatory mechanism of SIRT6‐mediated apoOBs clearance might hold great promise for bone maintenance and regeneration in the context of aging.

## 4 Experimental Section

### Mice

All experiments were performed with the approval of the Ethics Committee of the School of Stomatology of Nanjing Medical University. All procedures were carried out according to the guidelines of the Animal Care Committee of Nanjing Medical University (Approval No. IACUC‐2019293). Mice were raised on a 14/10 h light/dark cycle in the Animal Research Center of Nanjing Medical University. *LysMcre* (Cat# N000056) were from the Model Animal Research Center of Nanjing University and *SIRT6^f/f^* (JAX# 017334) and *Prx1cre* (JAX# 005584) mice were from Jackson Laboratory.^[^ ^63^, ^64^, ^65^ ^]^ To knockout SIRT6 specifically in Prx1‐lineage cells, *SIRT6^f/f^* mice were mated with *Prx1cre* mice to generate *Prx1cre;SIRT6^f/+^* heterozygous mice. Next, by crossing *Prx1cre;SIRT6^f/+^* and *SIRT6^f/f^* mice, *Prx1cre;SIRT6^f/f^* mice as homozygous conditional SIRT6 knockout mice were obtained. *LysMcre;SIRT6^f/f^* mice were obtained as the same process.

### Animal Experiments

For CD47 blockade treatment in vivo, a relatively low concentration of anti‐CD47 antibodies (2 mg k^−1^g body weight) was chosen to reduce potential hematotoxicity. Then, aged mice were then randomly divided into two groups and intrafemorally injected with anti‐CD47 antibodies or IgG (2 mg k^−1^g body weight) twice a month for 2 months. In the bone regeneration model, the first maxillary molars were extracted for tooth extraction based on the previous experience, and the maxillae were harvested after 2 weeks.^[^ ^66^ ^]^ For exosomes‐based delivery systems, exosomes were isolated from mouse vascular endothelial cells (KeyGEN BioTECH, Nanjing, China) and diluted in transfection buffer. The quercetin or OSS_128167 at a final concentration of 10 µm were added into 100 µL EC‐Exos sample and electroporated at 0.150 kV/100 µF under BTX Gemini X2 system. Drug‐loaded EC‐Exos were injected into mice twice per month via tail vein and after 3 months, femur samples were harvested and processed for further analysis (*n* = 6 per group).

### Cell Culture and NAD^+^ Measurement

Cell culture was described in previous studies.^[^ ^66^ ^]^ Briefly, for mouse apoptotic osteoblasts (apoOBs) culture, the bone tissue was cut into about 0.5 mm pieces, and then cultured with complete medium (DMEM, 100 U mL^−1^ penicillin and 100 µg mL^−1^ streptomycin, 10% FBS) for 3 days, at which point nonadherent cells were removed by replenishing culture medium. Adherent cells were passaged once reaching 80–90% confluence and after three passages, the bone marrow stromal cells (BMSCs) were obtained and then cultured in osteogenic medium for 3 days to induce into osteoblasts. apoOBs were obtained by introducing osteoblasts under ultraviolet light for 0.5 h (an irradiation‐induced apoptosis method).^[^ ^6^ ^]^ For macrophage cultures, bone marrow cells were flushed out from the femurs and tibias with DMEM and then seeded at 5 × 10^6^ onto 100 mm petri dishes for 4 h incubation. Next, nonadherent cells were collected and continuously cultured in complete DMEM supplemented with murine M‐CSF (10 ng mL^−1^) for 3 days. For macrophage polarization, the naive macrophages were stimulated with 100 ng mL^−1^ LPS for 2 days to induce inflammatory M1 phenotype and alternatively, cocultured with 20 ng mL^−1^ IL‐4 for 2 days to induce anti‐inflammatory M2 phenotype.

For NAD^+^ measurements, cells were resuspended and lysed with 200 µL NAD^+^/ NADH extract buffer and centrifuged at 12 000 g for 10 min on ice. The supernatant was collected for NAD^+^ measurements according to NAD^+^/NADH assay kit (Cat# S0175, Beyotime). NAD^+^ value was measured by calculating the value of total NAD^+^ and NADH (NAD^+^ = total NAD^+^‐NADH).

### Western Blot and Coimmunoprecipitation

Western blot was performed as described previously.^[^ ^66^ ^]^ Harvested cells were lysed on ice for 30 min with RIPA buffer (Cat# P0013B, Beyotime) containing 10 mm protease inhibitor. Protein lysate was separated onto 10–15% SDS‐PAGE gels and then transferred to an Immobilon‐PVDF membrane (Millipore, Billerica, MA). Membranes were blocked using 5% fat‐free milk at 22 °C and subsequently incubated with primary antibodies at 4 °C overnight. Detailed information on the primary antibodies is listed in Table S9 (Supporting Information). After washing with TBST three times, the membranes were incubated with secondary peroxidase‐conjugated antibodies for 1 h. Finally, the protein bands were visualized using an ECL detection kit (Millipore).

For co‐IP assay, cells or primary mouse MSCs were lysed in IP Lysate Buffer (P0013, Beyotime Biotechnology, Haimen, China) supplemented with 1% Halt Protease & Phosphatase Inhibitor Cocktail (78445, Thermo, USA) for 30 min. The supernatant was incubated with the specific antibody and added with 50 µL of protein A/G agarose beads (Roche, Mannheim, Germany) at 4 °C overnight. The beads were washed 3 times with 1 × washing buffer, resuspended in 1× SDS loading buffer and then subjected to Western blot assay. The antibodies used in this study are listed in Table S9 (Supporting Information).

### RT‐qPCR

Total RNA was extracted from cells using Trizol reagent and reverse transcribed using the PrimeScript RT Reagent Kit (Takara Bio, Kusatsu, Japan). miRNAs of purified apoVs were extracted using the miRNeasy Serum/Plasma kit (QIAGEN, Valencia, CA) according to the manufacturer's instructions. miRNAs expression were detected by the All‐in‐One miRNA RT‐qPCR Detection Kit (GeneCopoeia, Rockville, MD). The levels of each mRNA or miRNA were normalized to the GAPDH or U6 levels, respectively, while the miRNAs levels from apoVs were compared to the levels of spiked‐in ce‐miR‐39, which was applied as the reference using the miRNeasy Serum/Plasma Spike‐In Control kit (QIAGEN, Valencia, CA). Each experiment was performed in triplicate. The 2^−ΔΔCT^ method was used to quantify expression of the genes of interest. The primer sequences used in this study are listed in Table S8 (Supporting Information).

### ChIP‐Seq and ChIP‐qPCR Assays

For ChIP‐seq, OBs treated with PBS or OSS_128167 were cross‐linked with 1% formaldehyde for 10 min and quenched with 0.125 m glycine for 5 min. Then, cells were washed and resuspend in lysis buffer supplemented with protease inhibitors to obtain chromatin by centrifugation for 5 min at 800 rpm. Nuclear fraction was resuspended in 150 µL Nuclear Lysis Buffer (50 mm Tris‐HCl, pH 8.0, 10 mm EDTA, 1% SDS, protease inhibitors) for 10 min incubation. Genomic DNA was then sonicated and reverse‐crosslinked with 1 mL of proteinase K in elution buffer (50 mm NaHCO3, 140 mm NaCl, 1% SDS) at 65 C overnight. Chromatin was treated with RNase and phenol chloroform extraction and then measured on 1% agarose gel electrophoresis to select products with smear below 300 bp for following assay. Chromatin lysates were precleared and employed for immunoprecipitation (IP) with magnetic beads combined with 2 mg of anti‐Pol II (Rpb1 CTD, 4H8) (Cat# 2629) in IP buffer (20 mm Tris HCL pH8, 2 mm EDTA pH8, 150 mm NaCl, 0.1% SDS, 1% Triton x‐100) at 4 °C overnight. The complexes were pelleted and successively washed with IP buffer, TSE buffer (20 mm Tris HCL pH8, 2 mm EDTA pH8, 150 mm NaCl, 1% Triton x‐100, 0.1% SDS), and TE‐buffer (10 mm Tris‐HCL pH8.0, 1 mm EDTA) for 2 times. DNA was eluted with elution buffer (50 mm NaHCO3, 100 mm NaCl, 1% SDS) supplemented with 10 mg of proteinase K at 50 °C for 1 h. Then, DNA products were removed from the magnetic beads and reverse‐crosslinked at 65 °C for 4 h. The ChIP DNA was purified using a PCR purification column (QIAGEN Germany, Hilden). DNA libraries were constructed for DIP sequencing using Illumina reagents and sequencing equipment. Sequencing was performed using Illumina reagents and sequencing equipment to construct DNA libraries.

ChIP‐PCR assays were carried out according to the Hyperactive In Situ ChIP kit (Vazyme, Nanjing, China). Briefly, cells were collected and resuspended with concanavalin A coated magnetic beads (ConA beads) in PCR tube; subsequently, the binding cells to beads were immunoprecipitated with primary antibody overnight at 4 °C. Then, the tubes were placed on magnetic separator to pull off the supernatant. Beads were cocultured with secondary antibody (Bioworld) in Dig‐wash Buffer for 1 h and washed with dig‐wash buffer using magnetic separator to remove unbound antibodies. Next, hyperactive pG‐Tn5/pA‐Tn5 Transposon were added into tubes for 1 h with gentle vortexing and resuspended in 300 µL tagmentation buffer to generate DNA fragments. The tagmentation was stopped in a mixed liquid containing 10 µL 0.5 m EDTA, 3 µL 10% SDS, and 2.5 µL 20 mg mL^−1^ Proteinase K, and subsequently, the DNA were eluted by chloroform, ethanol, and Tris‐HCl. Finally, the purified DNA was ready for qPCR enrichment.

### ChIP‐Seq Analysis

Public ChIP‐seq raw data generated from wild type (WT) and SIRT6 knockout (KO) embryonic stem cells (ESCs), which were targeted by RNA Pol II, H3K9Ac, H3K56Ac, and LEO1 antibodies, were retrieved from the Gene Expression Omnibus (GEO) (GSE130689 and GSE65836). The ChIP‐seq raw data generated in OB and OSS_128167‐treated OBs using Pol II antibodies have been uploaded in GEO (GSE201651). Briefly, data were first quality controlled by FastQC v 0.11.5, and then, the adapters and low‐quality sequences were trimmed using Cutadapt 1.15. Subsequently, sequencing reads were uniquely aligned against the mm10 reference genome using BWA 0.7.12. BAM files, filtered using samblaster 0.1.19 to remove redundancy and duplicated sequences, were converted to bigWig files by deeptools 3.3.0. Signal tracks for each binding sites were predicted and generated by MACS2 2.1.2 and visualized by IGV 2.6.0.

### Efferocytosis and Flow Cytometry Assay

Before induced to apoptosis, osteoblasts were stained with CFSE (Cat# 65‐0850‐84, Invitrogen), a fluorescent membrane dye that can be easily measured. Then, apoOBs were recovered for 2 h in complete medium and cocultured with macrophage in equal number (1:1) for 3 h. After removing free apoOBs, cells were fixed with 4% formalin and macrophages were stained red (Cy3, F4/80). Efferocytic macrophages were captured as Cy3 and CFSE positive cells by confocal microscopy. To quantify efferocytic macrophages, cells were harvested and subjected to flow cytometry assay as in the previous study.^[^ ^67^ ^]^ Briefly, cocultured cells were treated with Fixation/Permeabilization Kit (Cat# 554715; BD Pharmingen) and stopped with Fc block reagent (Cat# 553141; BD Pharmingen). Then, macrophages were stained with PE rat antimouse F4/80 (Cat# 565410, BD Pharmingen). After removing free isotype control antibody, efferocytosis was verified as the percentage of double positive PE macrophages and CFSE‐stained apoOBs (FITC, apoOBs) by flow cytometry (BD Biosciences, San Jose, CA). For bone marrow staining, cells were first stained with DAPI to distinguish live cells and then FcR‐receptors were blocked with anti‐CD16/32 for 20 min (Cat# 101303; Biolegend). Subsequently, cells were performed for antibodies staining including: anti‐ALP (Cat# AF2910; R&D Systems), anti‐ anti‐Caspase‐9 (Cat# ab210611; Abcam), and anti‐CD47 (Cat# 127503; Biolegend). FlowJo v.10 software was used for the data analysis.

### Macrophage Recruitment and Migration Assay

Culture‐insert 2 well system (Cat# 80209, Ibidi) was used to assess the ability of apoOBs to recruit macrophages. Briefly, before induced to apoptosis, Culture‐insert 2 well were placed in the center of 12 well plates and CFSE‐stained osteoblasts immediately were seeded in the right well of the insert. Then, DiI‐labeled macrophages were seeded in the left well and the insert was removed to create a scratch after 3 h adherent of cells. The macrophages were recruited by apoVs across the scratch to phagocytose apoOBs. The migrated macrophages in scratch and right well of the insert were measured and analyzed by Image J. To further evaluate the effect of apoVs on macrophages recruitment, apoVs in 750 µL complete medium were added to the lower chamber and macrophages in 250 µL serum‐free medium were seeded into the upper chamber. After 24 h migration, the chambers were fixed and then stained with a 0.05% crystal violet solution for 15 min. Images were captured from five random fields under a light microscope and chosen for quantification.

### Isolation and Labeling of apoVs or EC‐Exos

To collect apoVs from the apoOBs, osteoblasts reaching ≈80–90% confluence were induced to apoptosis and cultured in serum‐free αMEM for 48 h. Then, the supernatant was harvested and centrifuged at 300×g for 10 min to remove cell lysate, and 2000×g for 15 min to remove cell debris. Subsequently, the apoVs were isolated by centrifugation at 100 000×g for 70 min twice, and the final pellet was resuspended with 100 µL PBS and stored at −80 °C. For EC‐Exos collection, the supernatant from vascular endothelial cells (ECs) following 48 h serum‐free culture was centrifuged at 300 g for 10 min, 2000 g for 15 min, and 10 000 g for 30 min, removing dead cells, cell debris, large EVs and collecting the supernatant each time. Then, the supernatant was centrifuged at 100 000 g for 70 min twice to discard the contaminating protein, and finally, EC‐Exos in pellet was resuspended with 100 µL PBS and stored at −80 °C.

To detect the uptake of apoVs by macrophages, osteoblasts were labeled with DiO cell‐labeling solution (Cat# 40725ES10, Yeasen) according to the manufacturer's protocol and induced to apoptosis. After 24 h incubation, the supernatant containing apoVs was collected and cocultured with macrophages at 37 °C for 2 h. Subsequently, cells were fixed with 4% paraformaldehyde, stained with DAPI, and captured under a fluorescence microscope (Leica Microsystems, Mannheim, Germany). To observe the distribution of EC‐Exos in the mouse, exosomes were labeled with DiI (Cat# 40726ES10, Yeasen) and intravenously injected into mice. After 4 and 8 h, the relevant organs were excised and analyzed by IVIS Spectrum (PerkinElmer, Waltham, MA).

### Micro Computed Tomography (micro‐CT) Analysis

The micro‐CT system (Skyscan 1176, Kontich, Belgium) was used to analyse microarchitectural properties of the distal femur and tooth extraction socket. Bones were scanned at a high resolution (15 µm) with an energy of 50 kV and 456 µA. NRecon v1.6 and CTAn v1.13.8.1 software were applied to reconstruct and analyze the 3D images of the bone. The region of interest (ROI) was defined as the distal femurs or tooth extraction socket. The ROI of trabecular morphometric traits started at the edge of the primary spongiosa and then proximally extended from this position for 200 slices with the boundary of endocortical margin. The ROI of cortical bone started at the end of trabecular bone ROI region and extended 10 slices toward the femoral head. To evaluate the trabecular and cortical bone structure, the following five parameters were calculated: the bone volume ratio (BV/TV, %), trabecular number (Tb.N.), trabecular separation (Tb.Sp.), bone mineral density (BMD), and cortical thickness (Ct. Th).

### rAAV9‐Transduced Gene Vectors Construction and Injection

Cre‐dependent recombinant AAVs (serotype rAAV9, vectors: CMV bGlobin‐FLEX‐MCS‐EGFP‐WPRE‐hGH polyA, Cat# AAV9‐49463‐1) were synthesized and acquired from GeneChem. Briefly, in the Cre‐dependent recombinant rAAV9 system, the gene flanked by the two reverse loxPs was in opposite directions, downstream of the promoter. In the Cre^+^ cells, rAAV9‐transduced gene can be turned on and overexpressed but silenced in the absence of Cre. Therefore, rAAV9‐transduced SIRT6 vectors can make SIRT6 overexpression in Prx1‐lineage cells or LysM‐lineage cells. Moreover, EGFP has been also inserted into rAAV9‐transduced gene vectors, thus leading to simultaneous SIRT6 and EGFP (tracking positively transduced cells) gene expression in the presence of Cre. *Prx1cre* and *LysMcre* mice (6‐months age) were injected with rAAV9‐SIRT6 (1.43×10^12^ virus genomes [v.g] mL^−1^) or control rAAV9 (5.2 × 10^12^ v.g mL^−1^) twice per month at a dose of 10 µL by periosteal injection into the marrow cavity of femur. Three months later, mice were euthanized and femur samples were harvested and processed for further analysis (*n* = 6 per group). Consistently, another Cre‐dependent recombinant AAVs (serotype rAAV9, vectors: CMV bGlobin‐FLEX‐EGFP‐MIR155(mcs)‐WPRE‐hGH polyA) were used to knock down CD47 in *Prx1cre;SIRT6^f/f^* mice. Then, rAAV9‐CD47 (4.97×10^13^ v.g mL^−1^) or control rAAV9 (1 × 10^13^ v.g mL^−1^) were injected into *Prx1cre;SIRT6^f/f^* mice twice per month for 2 months. The subsequent experiments were the same as before.

### Immunofluorescence, Histology, and Dynamic Histomorphometry Analysis

For immunofluorescence, bone tissue was fixed with 4% paraformaldehyde for 48 h, and decalcified in 10% EDTA for 6 weeks. The decalcified bones were immersed into 20% sucrose solution containing 2% polyvinylpyrrolidone for 24 h and then embedded in OCT for 15 µm thick slices. The slices were permeabilized with 1% Triton X‐100 for 15 min and incubated with goat serum to block nonspecific staining. Primary antibodies were incubated with bone tissue overnight at 4 °C. The antibodies used in immunofluorescence assay are listed in Table S9 (Supporting Information). After incubation with primary antibodies, slices were rinsed with PBS and incubated with FITC or Cy3‐labeled secondary IgG at 37 °C for 1 h. Finally, sections were labeled with DAPI and images were captured under fluorescence microscope (Leica Microsystems, Mannheim, Germany).

For histology, bone tissue structure was embedded in paraffin wax, sectioned into 4 µm thick slices and observed below the growth plate by masson trichrome staining or TRAP staining. The quantification of osteoblasts and osteoclasts were presented as a graph of the quantification of Ob. N/BS (osteoblast number per bone surface). Osteoclasts were quantified by Oc. N/BS (osteoclast number per bone surface).

To analyze the bone dynamics, the mice were administered a subcutaneous injection with 20 mg k^−1^g calcein (Sigma, USA) and 30 mg k^−1^g Alizarin red S (Sigma, USA) at 8 and 2 days, respectively, before euthanasia. Bone samples were embedded in methyl methacrylate, sectioned at 30 µm thin sections (EXAKT, Germany), and further thinned between 15 and 20 µm by abrasive paper. The images of trabecular bone were captured within a defined ROI that began 100 µm from the distal growth plate and extended to the diaphysis for 2500 µm. The cortical surface was captured in the defined ROI of the mid‐shaft of femur whose position was close to the ROI by micro‐CT and ≈150 µm of bone surface was measured for histomorphometry. The subsequent histomorphometric analysis of mineral apposition rate (MAR, µm/day), mineralizing surface/ bone surface (MS/BS) and bone formation rate per unit of bone surface (BFR/BS, µm^3^/µm^2^/day) were calculated based on recommendations of the Histomorphometry Nomenclature Committee of the American Society of Bone and Mineral Research and quantified by Image pro software to observe the mineralizing front.^[^ ^68^ ^]^

### Single‐Cell Analysis

Single‐cell RNA‐seq raw data was downloaded from NCBI Gene Expression Omnibus (GEO), labeling GSE138689. The scRNA‐seq data have been pre‐processed by the authors.^[^ ^30^ ^]^ They excluded cells with fewer than 500 detected genes, more than 15% mitochondrial transcripts, more than 2% hemoglobin transcripts and contaminating endothelial cells. The log‐transformed digital matrix was performed for downstream analyses. To analyze 5022 LepR‐Cre^+^ cells from young mice (8‐week‐old) and 2619 LepR‐Cre^+^ cells from aged mice (12‐month‐old), the Seurat package (v3.0.1) was used to operate dimensionality reduction and clustering.^[^ ^30^ ^]^ After processing with principal component analysis (PCA) using the Jack Straw and graph‐based clustering using Louvain Method, the 2D t‐distributed stochastic neighbor embedding (t‐SNE) map were obtained.^[^ ^69^ ^]^ Next, to perform cluster specific markers for each cluster, Find Markers in Seurat for differentially expressed genes (DEG) analysis was used.^[^ ^70^ ^]^ The DEGs of OLCs (6.33% in young groups; 3.89% in aged groups) that were detected with a log2 fold change > 0.2 compared with all other clusters were considered to be marker genes. Monocle V2 package (v2.10.0) were used to describe the developmental process of OLCs and sort them in pseudotime.^[^ ^71^ ^]^ DEGs in each cluster were used to order genes to construct differentiation trajectories. To perform Gene Ontology (GO) analysis, the clusterProfiler package was used to analyze the DEGs in each cluster, and the results were filtered by setting a *p*‐value cutoff of 0.05.^[^ ^72^ ^]^

### RNA‐Seq Analysis

The quality of the RNA for sequencing was determined using agarose gel electrophoresis and Nanodrop. RNA library was prepared using U‐mRNAseq Library Pre Kit and then profiled on Illumina Novaseq 6000 based on the method of sequencing by synthesis. The quantitative analysis of RNA‐seq data were used by HTSeq software and differential gene expression was determined by edgeR software. Differential gene expression of CD47 in young and osteoporotic osteoblasts (GSE35956/35958/35959/156508/80614/84500) and correlation analyses of osteoporotic patients (GSE35959/35956/156508/35958/10311) from public data were performed on the webpage of “home for researchers” (https://www.home‐for‐researchers.com ^−1^static/index.html#/).

### LC‐MS/MS Data Analysis

For data analysis, raw MS files from the vendors were analyzed using the Proteome Discoverer (PD) software (Version 2.4.0.305) and the built‐in Sequest HT search engine. MS spectra lists were searched against their species‐level UniProt FASTA databases. Trypsin was served as proteases. The peptide tolerance was set to 10 ppm and MS/MS tolerance was 0.02 Da. The false discovery rate (FDR) was set to 0.01 for both peptide levels and PSM, and a maximum of 2 missed cleavage(s) was permitted. Razor peptide and Unique peptide were utilized for total peptide amount for normalization and protein quantification. All the other parameters were reserved as default.

### miRNA‐Seq Analysis

After sequencing, raw reads were filtered using fastp software to remove low quality reads and residual Illumina adapters. After clipping, miRdeep2 software was used to align known miRNAs, allowing for 1 base mismatch. miRNA expression corresponding to its reads were counted and calculated using edgeR software, whose significant differentially expression were identified by fold change, *p*‐value and FDR between the two groups. miRNA targets were predicted by Miranda and TargetScan, and picked out based on the overlapping results of two databases. The predicted genes were carried out by clusterProfiler software for Gene Ontology (GO) and Kyoto Encyclopedia of Genes and Genomes (KEGG) Pathway analysis. The top 10 differentially enriched GO functions and KEGG pathways are represented as bar or dot plots.

### Statistical Analysis

All the data are expressed as the means ± SD. Experiments were independently repeated at least three times. Statistical analysis was performed with GraphPad Prism software (v9.3.0 for MacOS). Statistical significance of two groups comparison was assessed using Student's *t*‐test. Analysis across multiple comparisons was performed for one‐way ANOVA. *P* < 0.05 (*), *p* < 0.01 (**), and *p* < 0.001 (***) was considered statistically significant.

Additional methodological details are provided in the Materials and Methods of the Supporting Information.

## Conflict of Interest

The authors declare no conflict of interest.

## Author Contributions

R.X. performed experiments, analyzed data, and wrote the manuscript. H.X. and X.S. assisted R.X. with bioinformatics analysis, animal studies and histology. J.H., H.Z., and Y.F. collected clinical samples and analyzed the data, S.G., P.Z., and D.W. performed bioinformatics and statistical analyses. J.C., W.S., K.Z., and L.L. helped design experiments. H.J. conceived of this study, analyzed the data, and revised the manuscript. All authors read and approved the final manuscript.

## Supporting information

Supporting Information

Supplemental Table 1

Supplemental Table 2

Supplemental Table 3

Supplemental Table 4

Supplemental Table 5

Supplemental Table 6

Supplemental Table 7

Supplemental Table 8

Supplemental Table 9

## Data Availability

The data that support the findings of this study are available in the Supporting Information of this article.
