# Supplementary material for: Impaired Efferocytosis Enables Apoptotic Osteoblasts to Escape Osteoimmune Surveillance During Aging
Source: Adv Sci (Weinh). 2023 Oct 28;10(36):2303946. doi: 10.1002/advs.202303946 (PMC10754079; doi:10.1002/advs.202303946)
Supplement: Supplementary file 1 — Supporting Information [file ADVS-10-2303946-s005.pdf]

ADVANCED SCIENCE
Open Access

Supporting Information

for *Adv. Sci.*, DOI 10.1002/advs.202303946

Impaired Efferocytosis Enables Apoptotic Osteoblasts to Escape Osteoimmune Surveillance During Aging

Rongyao Xu, Hanyu Xie, Xin Shen, Jiadong Huang, Hengguo Zhang, Yu Fu, Ping Zhang, Songsong Guo, Dongmiao Wang, Sheng Li, Kai Zheng, Wen Sun, Laikui Liu, Jie Cheng and Hongbing Jiang**

Supplementary Figures for

Impaired efferocytosis enables apoptotic osteoblasts to escape osteoimmune surveillance during aging

Authors: Rongyao Xu[1,2,4], Hanyu Xie[1,2,4], Xin Shen[1,2,4], Jiadong Huang[1,4], Hengguo Zhang[1,4], Yu Fu[1,2,4], Ping Zhang[1,2,4], Songsong Guo[1,2,4], Dongmiao Wang[1,2,4], Sheng Li[1,2,4], Kai Zheng[1,4], Wen Sun[1,3,4], Laikui Liu[1,3,4], Jie Cheng[1,2,4*], Hongbing Jiang[1,2,4*]

Affiliations:

[1]Jiangsu Key Laboratory of Oral Diseases, Nanjing Medical University, Nanjing 210029, Jiangsu Province, China

[2]Department of Oral and Maxillofacial Surgery, Affiliated Hospital of Stomatology, Nanjing Medical University, Nanjing 210029, Jiangsu Province, China

[3]Department of Basic Science of Stomatology, Affiliated Hospital of Stomatology, Nanjing Medical University, Nanjing, Jiangsu 211166, China

[4]Jiangsu Province Engineering Research Center of Stomatological Translational Medicine, Nanjing 210029, Jiangsu Province, China

[*]Corresponding author:

Hongbing Jiang, D.D.S, Ph.D. E-mail: jhb@njmu.edu.cn

Jie Cheng, Ph.D. E-mail: leonardo_cheng@163.com

Jiangsu Key Laboratory of Oral Diseases, Nanjing Medical University, 140 Hanzhong Road, Nanjing, Jiangsu Province 210029, China.

Tel: +86-25-85031914

Fax: +86-25-85031910

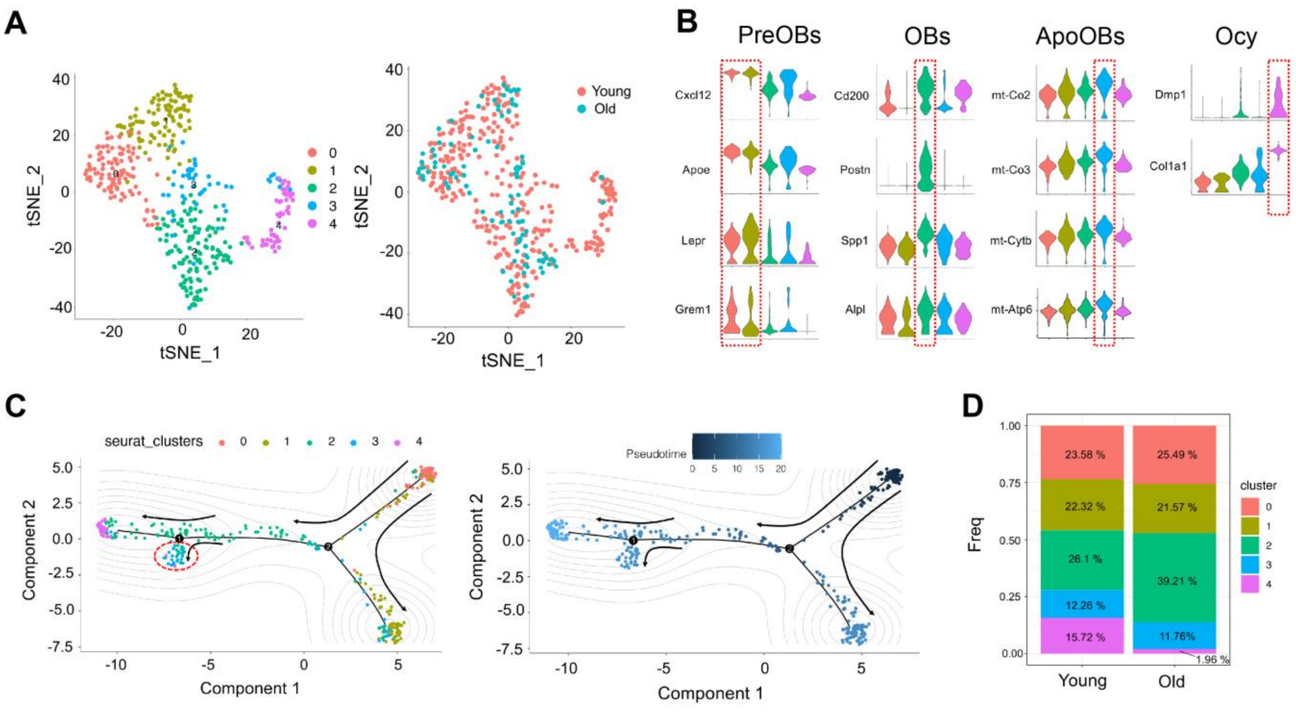

Supplementary Figure 1. scRNA-seq analyses of LepR-Cre-traced cells from young and old bone marrow. (A) The tSNE plots of osteoblast lineage cells isolated from young and old bone marrow. **(B)** Violin plots of marker gene expression for Pre-OBs (cluster 0,1), OBs (cluster 2), apoOBs (cluster 3) and osteocytes (Ocy, cluster 4) clusters. **(C)** Monocle trajectory plot of osteoblast lineage cells (OLCs) with the inferred direction of differentiation indicated by arrow. **(D)** Quantitative proportions of different clusters in young and aged OLCs.

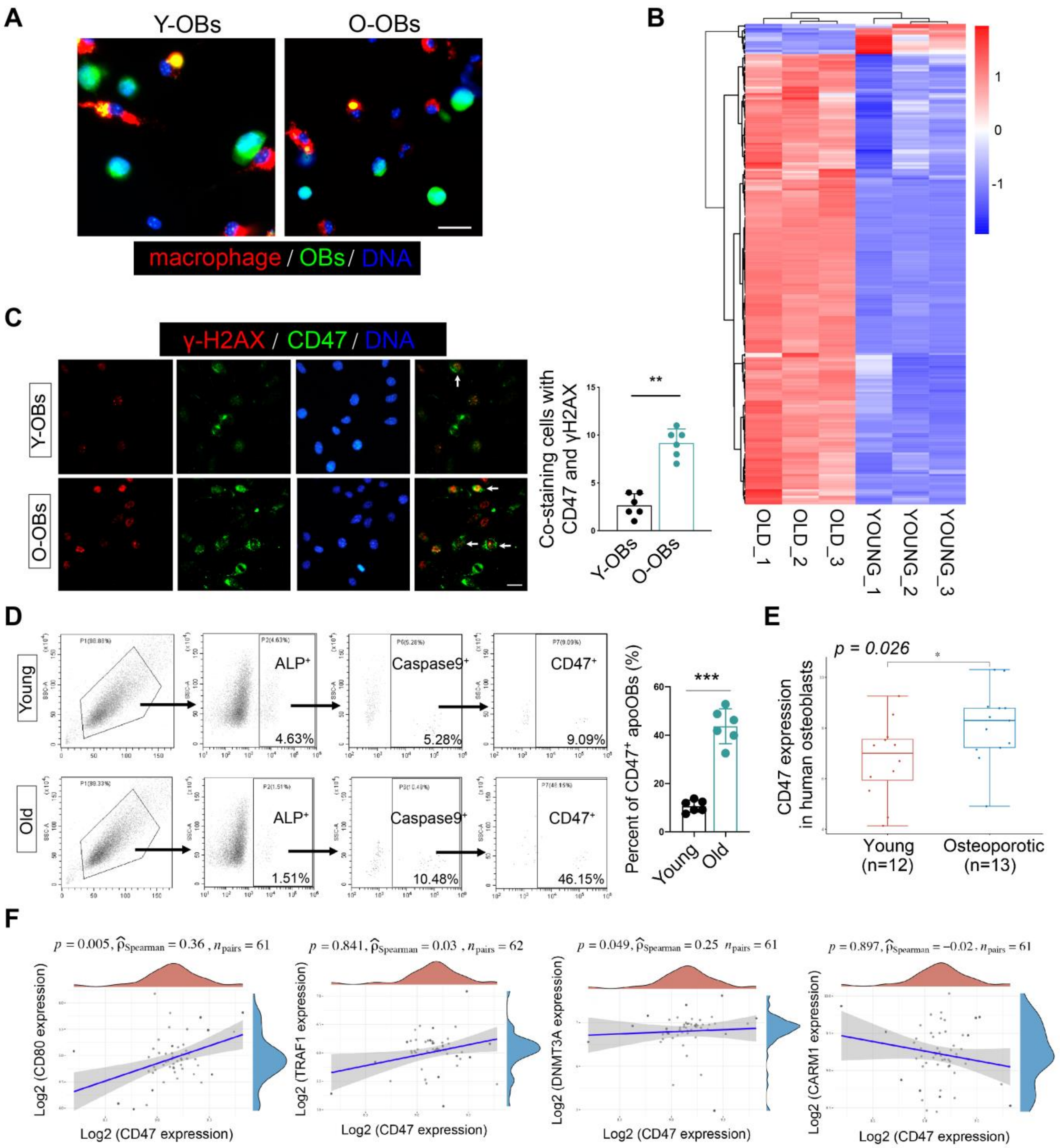

Supplementary Figure 2. Increased CD47 expression with aging. (A) Phagocytosis assay of osteoblasts (OBs) efferocytosis from young and old mice. Scale bars = 20 μm. **(B)** Heatmap revealing the gene expression profiles of apoOBs from young and old mice by RNA-seq (n=3). **(C)** Immunofluorescence staining for γH2AX and CD47 in OBs from young and old mice. Right panel showed the quantitative analysis. Scale bars = 50 μm. **(D)** Gating strategy to isolate and compare the difference of ALP^+ $Caspase9^+$ $CD47^+$cells in young and aged bone marrow. Right panel showed the quantitative data (n=6). **(E)** Analysis of differential gene expression of CD47 in young and osteoporotic osteoblasts based on public database. **(F)** Analysis of the correlation between CD47 and CD80, TRAF1, DNMT3A and CARM1 in osteoporotic patients based on public database. Results are presented as the mean ± S.D. **p* < 0.05; ***p* < 0.01; ****p* < 0.001 by unpaired 2-tailed Student's t test.

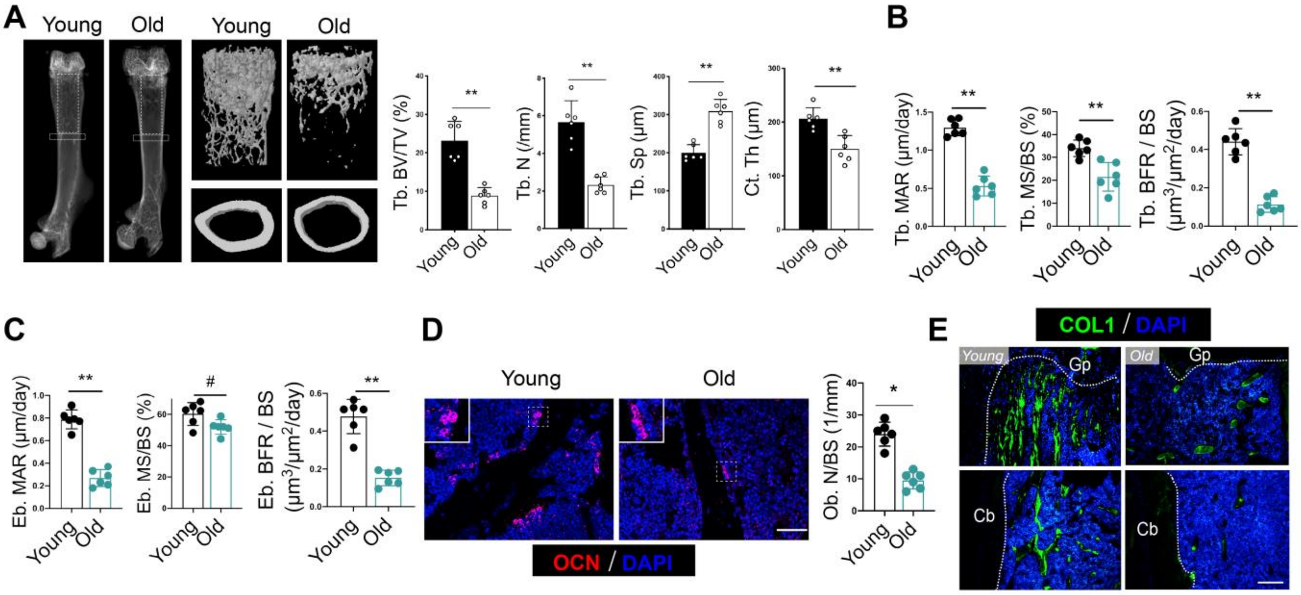

Supplementary Figure 3. Decreased bone mass in aged mice. (A) Representative micro-CT images trabecular and cortical bone and quantitative measurements of Tb.BV/TV, Tb.N, Tb.Sp and Ct.Th in young and old mice. **(B-C)** Quantitative measurements of dynamic histomorphometry of trabecular bone (Tb) and endosteal bone (Eb) of mineralization apposition rate (MAR), mineralizing surface/bone surface (MS/BS) and bone formation rate per bone surface (BFR/BS). **(D)** Representative immunofluorescence staining images of OCN and quantitative for Ob.N/BS analysis. Scale bars = 50 μm. **(E)** Immunofluorescence assay of $COL1^+$ area on trabecular and cortical bone surface. Gp, growth plate. Cb, cortical bone. Scale bar = 100 μm. Results are presented as the mean ± S.D. *p < 0.05; **p < 0.01; #p > 0.05 by unpaired 2-tailed Student's t test.

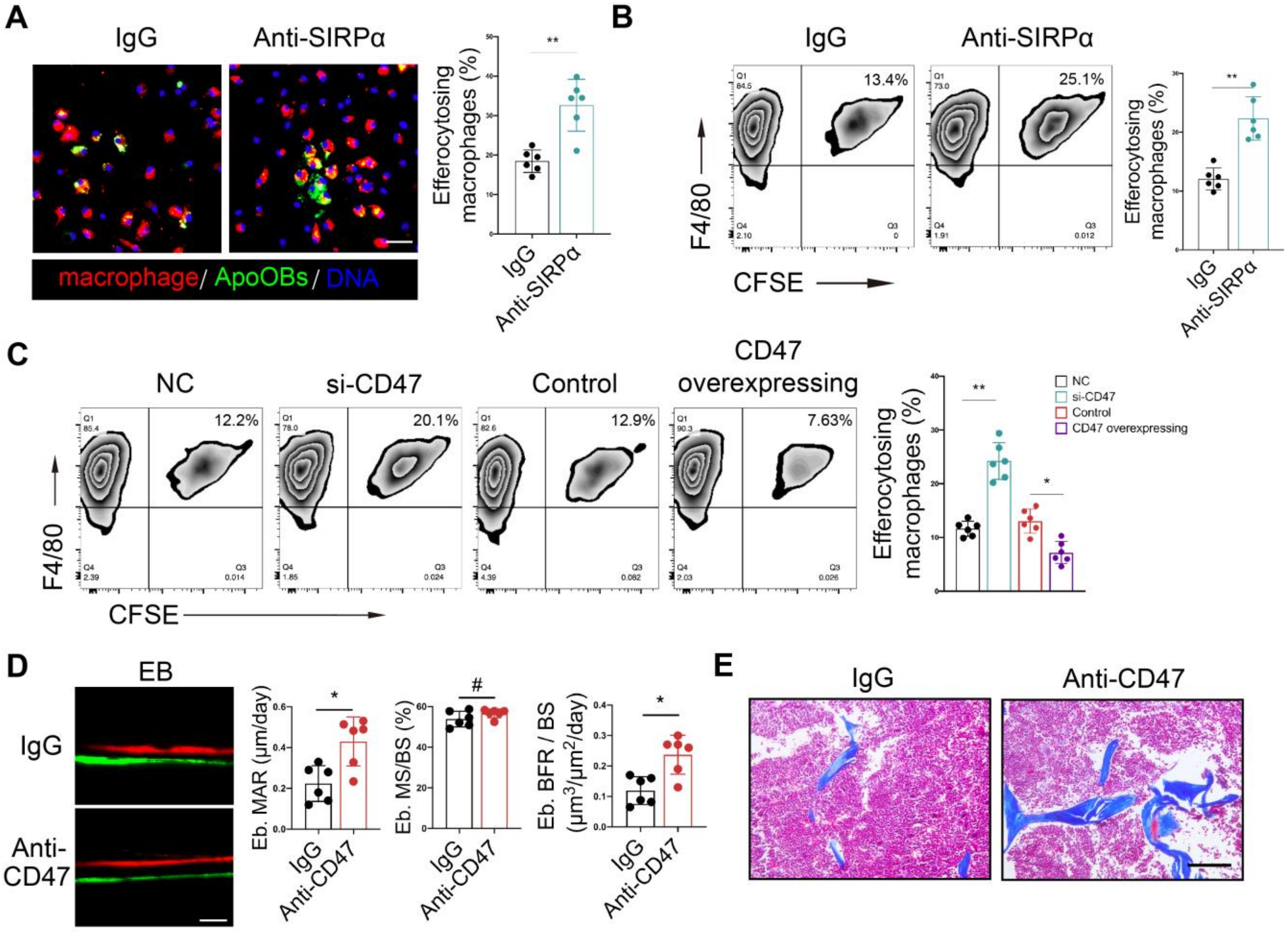

Supplementary Figure 4. SIRPα blockade strengthens apoOBs clearance and injection of anti-CD47 antibodies reduces age-related bone loss. (A) Phagocytosis assay. engulfment of apoOBs (green) by macrophages (red) with blocking control IgG or anti-SIRPα antibodies by immunofluorescence. Right panel showed the quantitative data (n=6). Scale bars = 20 µm. **(B)** Flow cytometric analysis of efferocytosis following blockade with anti-SIRPα antibodies with quantitative analysis at right panel. **(C)** Flow cytometric analysis showed that knockdown of CD47 increased apoOBs clearance, whereas overexpression of CD47 decreased the efferocytosis. Right panel showed the quantitative data (n=6). **(D)** Representative images of dynamic histomorphometry of endosteal bone (Eb) with quantification of mineralization apposition rate (MAR), mineralizing surface/bone surface (MS/BS) and bone formation rate per unit of bone surface (BFR/BS). Scale bars = 20 µm. **(E)** Representative masson staining images of trabecular bone mass in animals with CD47 blockade or IgG treatment. Scale bars = 50 µm. Results are presented as the mean ± S.D. *$p < 0.05$; **$p < 0.01$; #$p > 0.05$ by unpaired 2-tailed Student's t test.

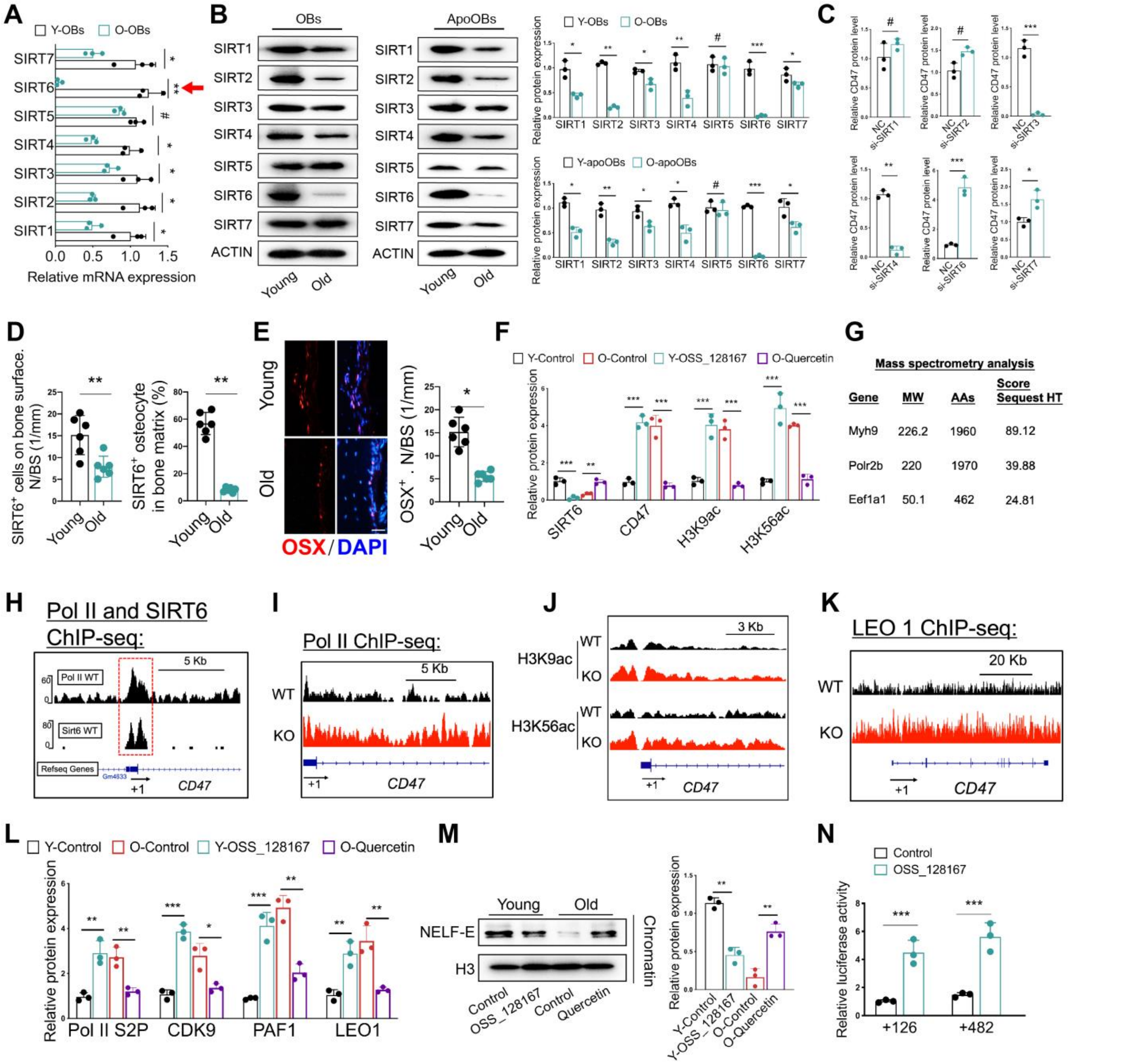

Supplementary Figure 5. SIRT6 retards CD47 expression in apoOBs through transcriptional pausing. **(A)** Quantitative qPCR analysis of the mRNA levels of sirtuins in OBs (n=3). **(B)** Western blot analysis of the protein levels of sirtuins in OBs or apoOBs. Right panel: Quantification of protein levels (n=3). **(C)** Quantification of CD47 protein levels in apoOBs treated with siRNA SIRT1, 2, 3, 4, 6 or 7 (n=3). **(D)** Quantitative measurements of SIRT6 in bone surface and matrix (n=6). **(E)** Immunofluorescence assay of OSX^+ cells on bone surface (n=6). Scale bars: 10 μm. **(F)** Quantification of SIRT6, CD47, H3K9ac, H3K56ac proteins in apoOBs with aging or SIRT6 pharmacological interventions (n=3). **(G)** Mass spectrometry analysis in apoOBs from young mice showing the enrichment for Myh9, Polr2b and Eef1a1. **(H)** Analysis of Pol II and SIRT6 ChIP-seq showing co-localization between Pol II and SIRT6 on CD47 promoter-proximal sites. **(I)** IGV browser images for Pol II ChIP-seq in WT and SIRT6 KO cells displaying elevated occupation on Pol II at intragenic regions of CD47. **(J)** Upregulated H3K9ac and H3K56ac levels in SIRT6 KO cells at intragenic regions. **(K)** IGV browser images from LEO1 ChIP-seq displaying increased recruitment of LEO1 at CD47 gene bodies. **(L)** Quantitative measurements of Pol II S2P, CDK9, PAF1 and LEO1 proteins in apoOBs with aging or SIRT6 pharmacological interventions (n=3). **(M)** Protein levels and quantitative measurements of NELF-E in chromatin fraction of apoOBs (n=3). **(N)** The predicted promoter-proximal regions were cloned

in luciferase reporters and luciferase activity in 293T cells were co-transfected with PBS or OSS_128167 (n=3). Results are presented as the mean ± S.D. *$p < 0.05$; **$p < 0.01$; ***$p < 0.001$; #$p > 0.05$ by one-way ANOVA followed with Tukey multiple comparisons tests or unpaired 2-tailed Student's t test.

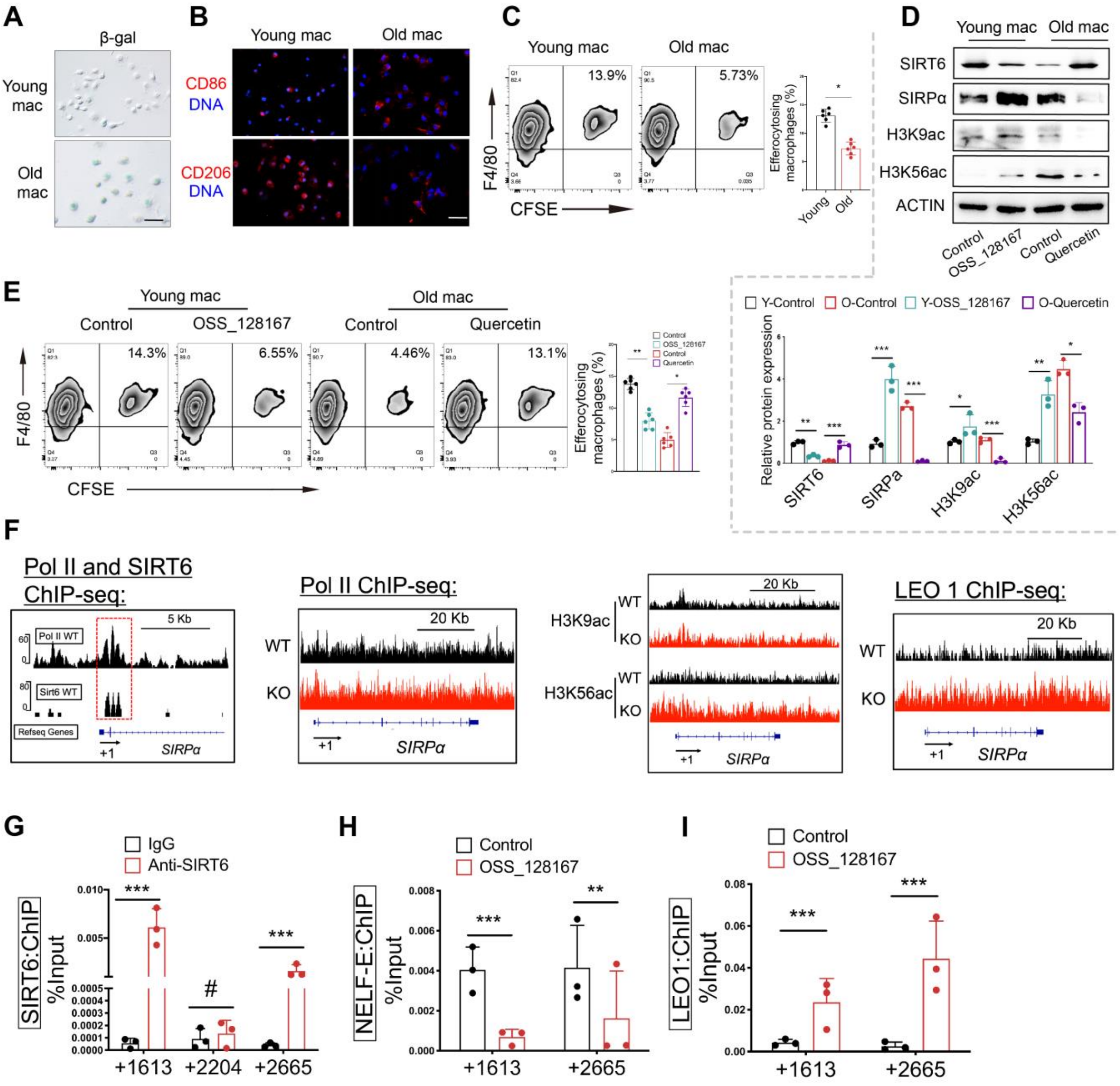

Supplementary Figure 6. SIRT6 regulates the engulfment ability of macrophages by affecting SIRPα expression. (A) SA-β-gal staining showing the senescence difference in young and aged macrophages. Scale bars = 50 μm. **(B)** The difference of macrophage polarization in response with aging was detected with anti–CD86 and anti–CD206 antibodies. Scale bars = 50 μm. **(C)** Flow cytometric analysis for the percentage of macrophages that efferocytosed apoOBs with quantitative measurements at right panel (n=6). **(D)** Proteins of SIRT6, SIRPα, H3K9ac and H3K56ac were analyzed by Western blot with quantitative measurements beneath (n=3). **(E)** Phagocytosis assay for macrophage efferocytosis with SIRT6 alternation. Right panel showed the quantitative data (n=6). **(F)** Analysis of Pol II, SIRT6, H3K9ac, H3K56ac and LEO1 ChIP-seq on SIRPα promoter-proximal sites. **(G-I)** ChIP-qPCR for SIRT6, NELF-E and LEO1 on SIRPα genes in primary macrophages (n=3). Results are presented as the mean ± S.D. *$p < 0.05$; **$p < 0.01$; ***$p < 0.001$; #$p > 0.05$ by one-way ANOVA followed with Tukey multiple comparisons tests or unpaired 2-tailed Student's t test.

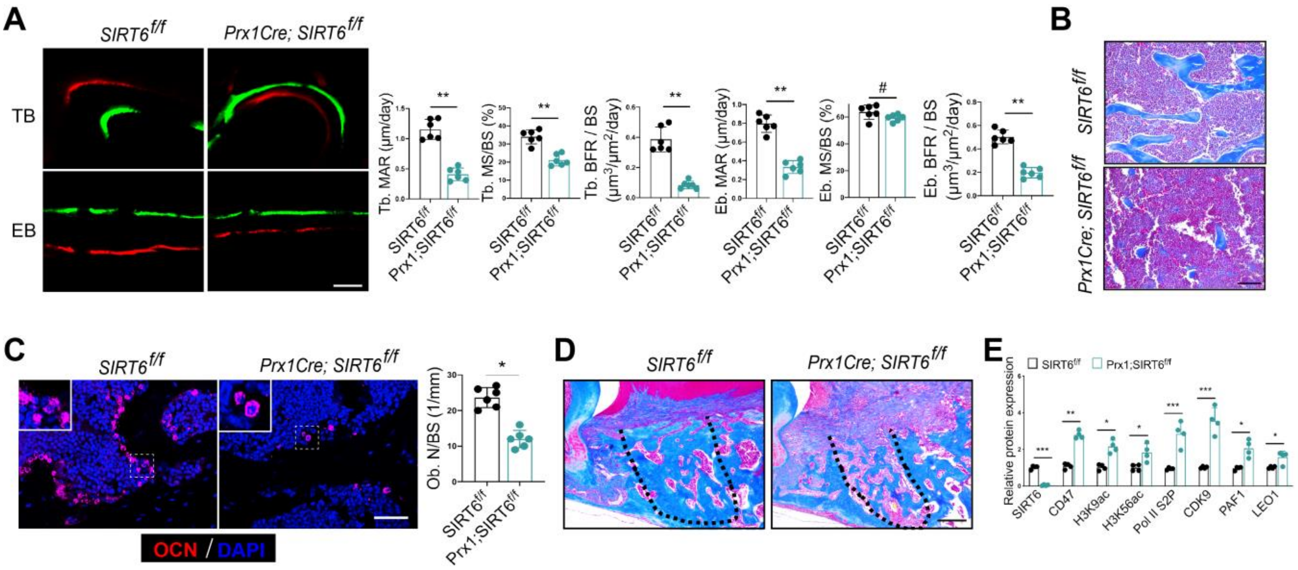

Supplementary Figure 7. Deletion of SIRT6 in osteoblast progenitors skeletal development and defect bone regeneration. (A) Representative images of dual labeling of Tb and Eb with quantification of MAR, MS/BS and BFR/BS (n=6). Scale bars = 20 µm. **(B)** Representative masson trichrome staining showing the difference of trabecular bone mass (n=6). Scale bars = 100 µm. **(C)** Representative immunofluorescence staining images of OCN and quantitative for Ob.N/BS analysis (n=6). Scale bars = 50 µm. **(D)** Representative masson trichrome staining showing the newly trabecular bone in tooth extraction socket. Black dotted line indicated tooth extraction socket (n=6). Scale bar = 200 µm. **(E)** Quantification of SIRT6, CD47, H3K9ac, H3K56ac, Pol II, Pol II S2P, CDK9, PAF1 and LEO1 proteins in apoOBs from *Prx1cre;SIRT6$^{f/f}$* and *SIRT6$^{f/f}$* mice (n=4). Results are presented as the mean ± S.D. **p* < 0.05; ***p* < 0.01; ****p* < 0.001; #*p* > 0.05 by unpaired 2-tailed Student's t test.

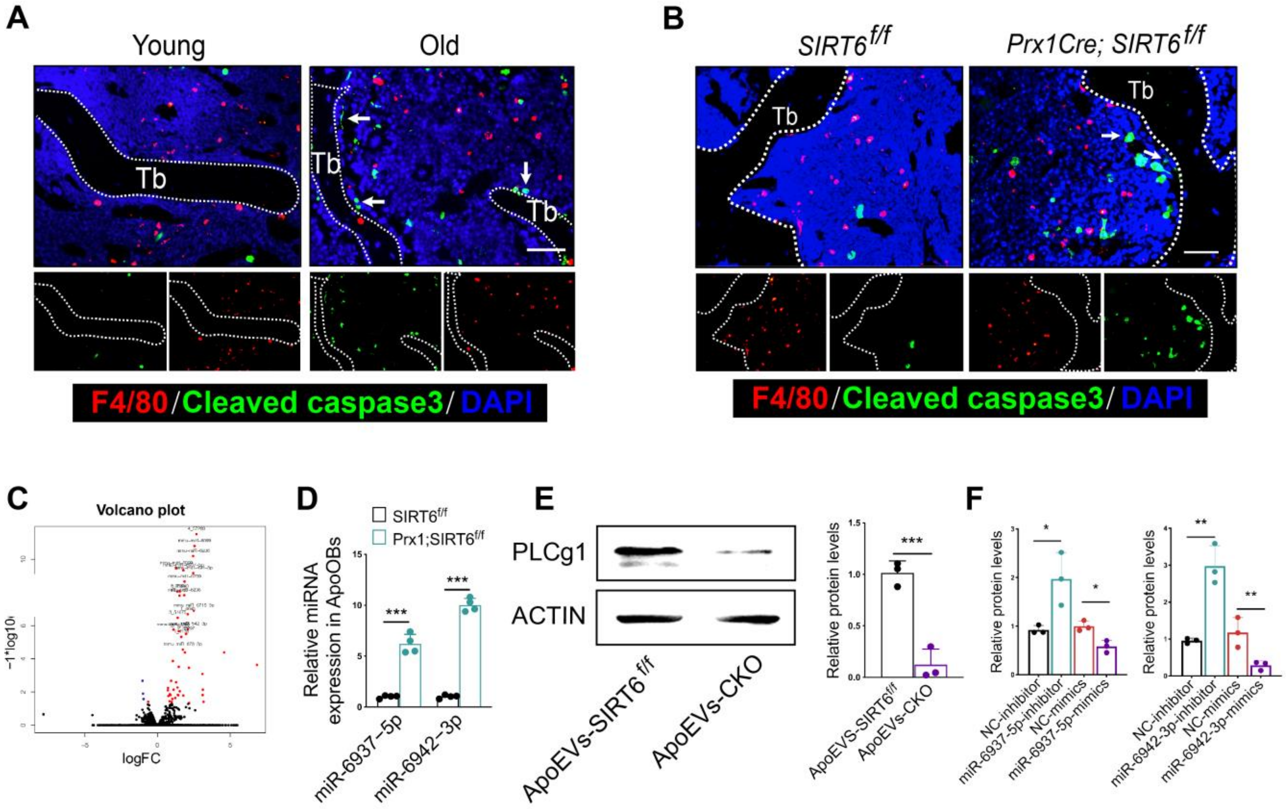

Supplementary Figure 8. ApoOBs regulates macrophages recruitment by releasing miRNAs loaded apoEVs. (A) Representative images of cleaved caspase3 and F4/80 immunostaining in young and old mice. Arrow denotes apoOBs. Scale bar = 50 μm. **(B)** Representative images of cleaved caspase3 and F4/80 immunostaining in *SIRT6*$^{f/f}$ and *Prx1cre;SIRT6*$^{f/f}$ mice. Scale bar = 50 μm. **(C)** Volcano plot showing that most differential miRNAs were upregulated. **(D)** The miR-6937-5p and miR-6942-53p loaded in apoOBs from *SIRT6*$^{f/f}$ and *Prx1cre;SIRT6*$^{f/f}$ mice were examined by qRT-PCR. **(E)** Western blot showing the PLCg1 expression in macrophages treated with apoEVs from *SIRT6*$^{f/f}$ and *Prx1cre;SIRT6*$^{f/f}$ mice. Right panel: quantitative measurements. **(F)** Quantification of PLCg1 proteins in miR-6937-5p and miR-6942-53p inhibitor or mimics treated macrophages. (n=3). Results are presented as the mean ± S.D. *$p < 0.05$; **$p < 0.01$; ***$p < 0.001$ by one-way ANOVA followed with Tukey multiple comparisons tests or unpaired 2-tailed Student's t test.

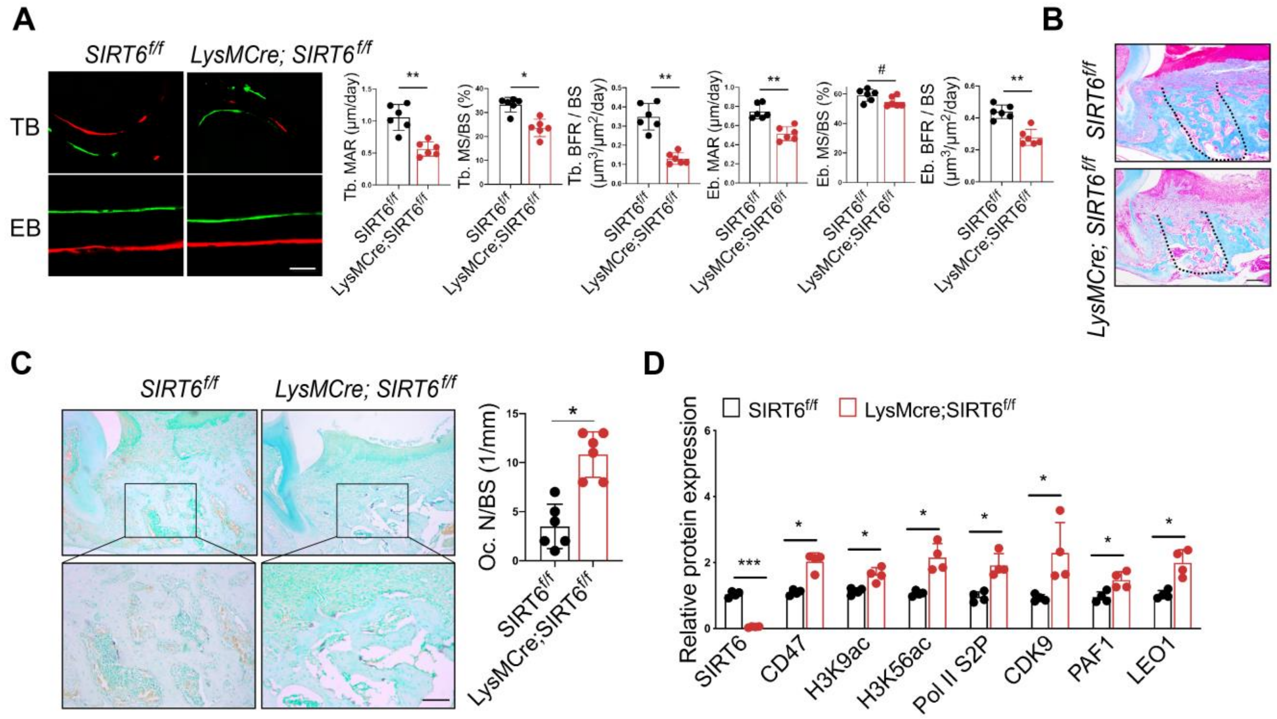

Supplementary Figure 9. SIRT6 deficiency in macrophages resulted in increased bone loss. (A) Representative images of dual labeling of Tb and Eb with quantification of MAR, MS/BS and BFR/BS (n=6). Scale bars = 20 μm. **(B)** Representative masson trichrome staining showing the newly trabecular bone in tooth extraction socket. Black dotted line indicated tooth extraction socket (n=6). Scale bar = 200 μm. **(C)** Representative TRAP staining images of extraction socket in *LysMcre;SIRT6$^{f/f}$* and *SIRT6$^{f/f}$* mice and quantification of osteoclasts per bone surface (n=6). Scale bar = 100 μm. **(D)** Quantification of SIRT6, CD47, H3K9ac, H3K56ac, Pol II, Pol II S2P, CDK9, PAF1 and LEO1 proteins in macrophages from *LysMcre;SIRT6$^{f/f}$* and *SIRT6$^{f/f}$* mice (n=4). Results are presented as the mean ± S.D. **p* < 0.05; ***p* < 0.01; ****p* < 0.001; #*p* > 0.05 by unpaired 2-tailed Student's t test.

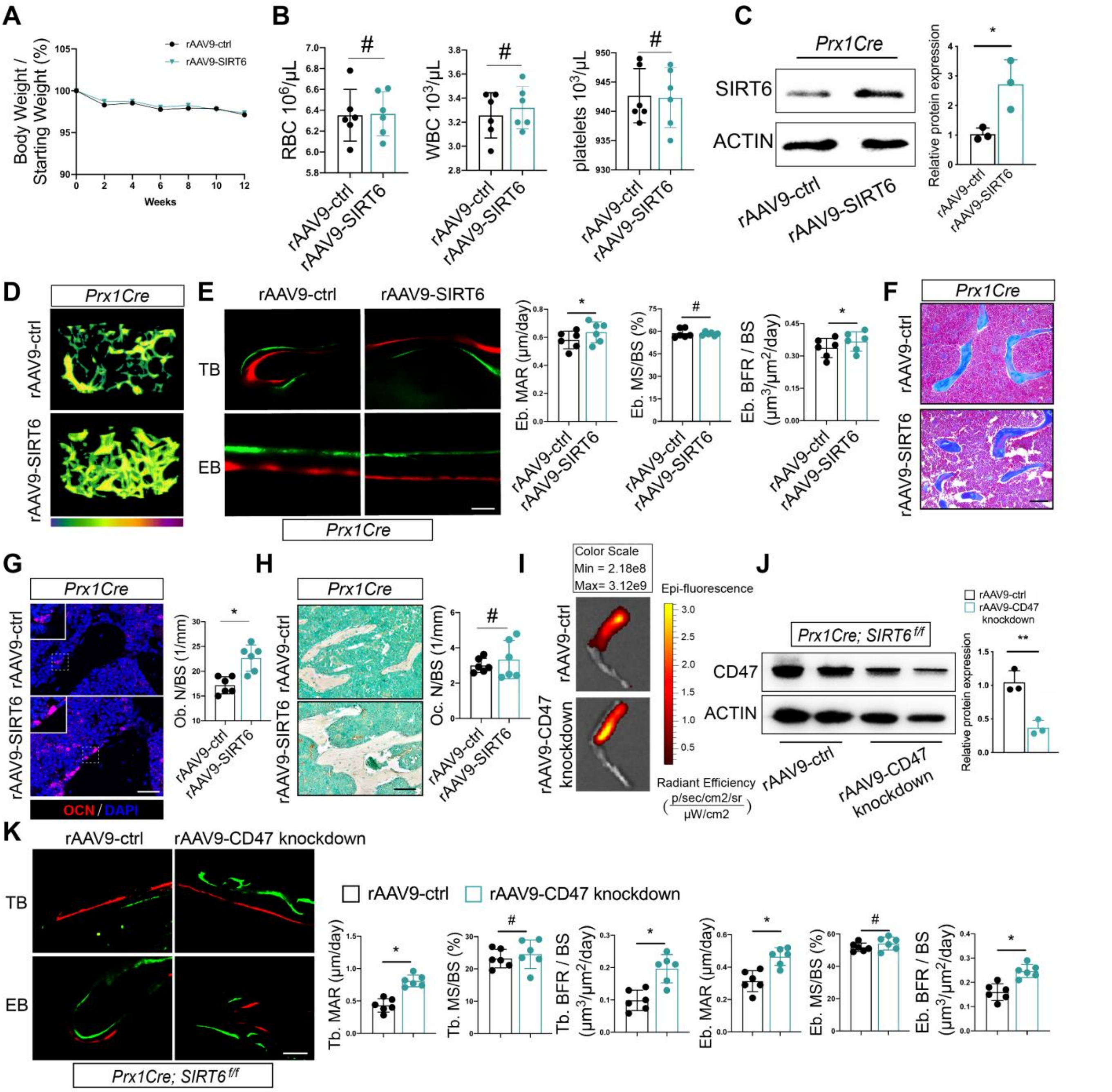

Supplementary Figure 10. Endogenously overexpressing SIRT6 increases bone accrual in *Prx1cre* mice. **(A)** Calculating the body weights by normalizing the starting body weight between rAAV9-control- and rAAV9-SIRT6-treated *Prx1cre* mice. **(B)** Total white cell counts, red cell counts and platelets counts were observed in each mouse. **(C)** After operation, osteoblasts were harvested for the validation of overexpression efficiency of SIRT6 by Western blot with quantitative measurements at right. **(D)** The trabecular thickness of trabecular bone was color coded in rAAV9-control- and rAAV9-SIRT6-treated *Prx1cre* mice. Blue-green color, thinner trabeculae; yellow-red color, thicker trabeculae. **(E)** Representative calcein/alizarin red labeling of Tb and Eb in rAAV9-control- and rAAV9-SIRT6-treated *Prx1cre* mice. Right panel, quantification of Eb with MAR, MS/BS and BFR/BS. Scale bars = 20 μm. **(F)** Femoral trabecular bone mass in rAAV9-control- and rAAV9-SIRT6-treated *Prx1cre* mice were assessed by masson trichrome staining. Scale bars = 100 μm. **(G)** OCN staining images and quantitative data for Ob.N/BS analysis (n=6). Scale bars = 50 μm. **(H)** Representative TRAP staining images of extraction socket in rAAV9-control- and rAAV9-SIRT6-treated *Prx1cre* mice and quantification of osteoclasts per bone surface (n=6). Scale bar = 50 μm. **(I)** rAAV9-CD47 knockdown and rAAV9-control were intrafemorally injected into bone marrow cavity of 3-month-old

Prx1cre;SIRT6$^{f/f}$ mice by IVIS optical imaging. Y-axis indicates radiant efficiency (p/s/cm2/sr/µW/cm2). **(J)** After injection of rAAV9-CD47 knockdown, osteoblasts were harvested in bone marrow from *Prx1cre;SIRT6*$^{f/f}$ mice for validating knockdown efficiency of CD47 by Western blot. Quantitative measurements at right. **(K)** Representative images of dual labeling of Tb and Eb with quantification of MAR, MS/BS and BFR/BS (n=6). Scale bars = 20 µm. Results are presented as the mean ± S.D. **p* < 0.05; ***p* < 0.01; #*p* > 0.05 by unpaired 2-tailed Student's t test.

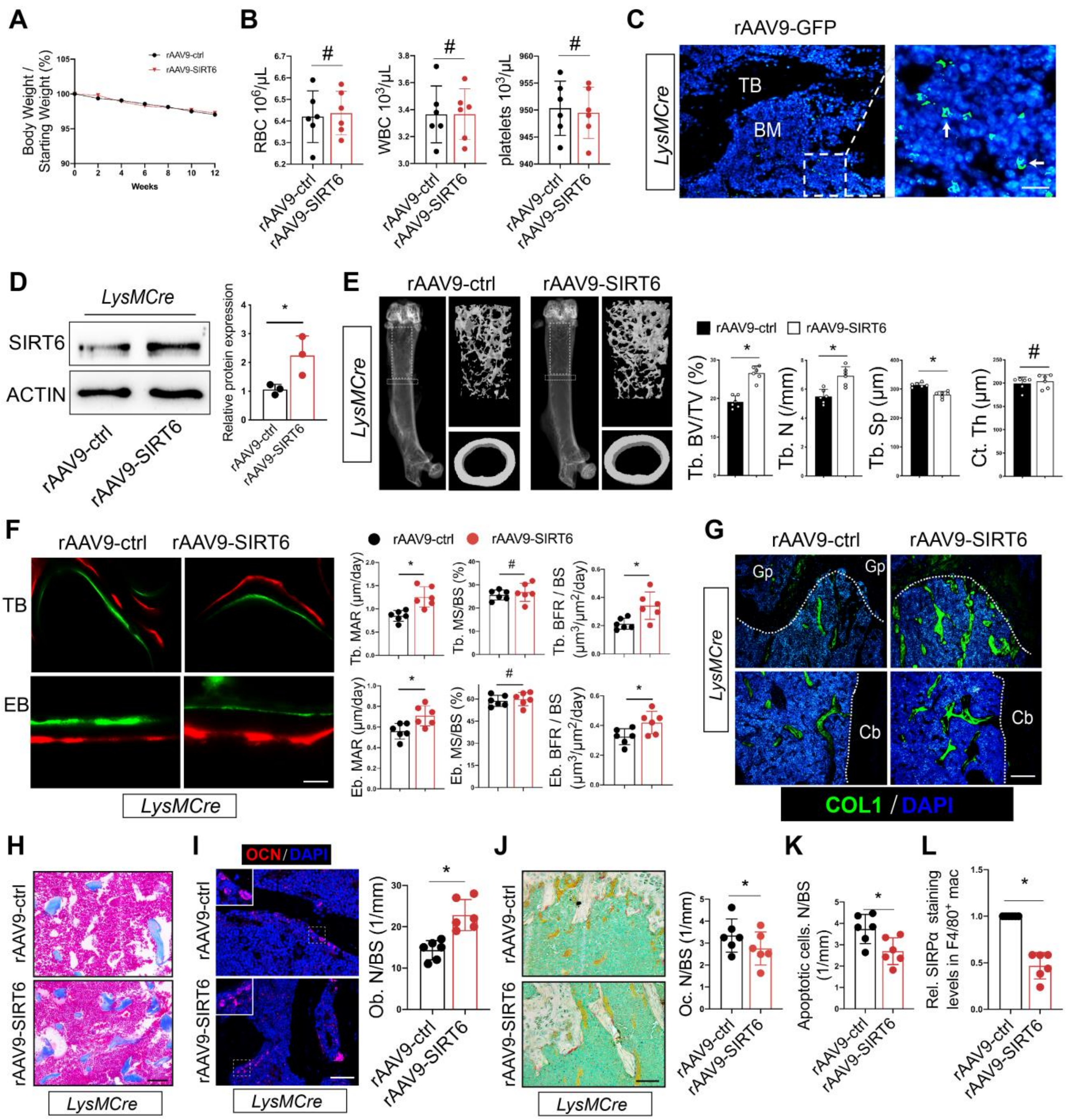

Supplementary Figure 11. Endogenously overexpressing SIRT6 increases bone mass in *LysMcre* mice. **(A)** Normalized body weights between rAAV9-control- and rAAV9-SIRT6-treated *LysMcre* mice. **(B)** Total white cell counts, red cell counts and platelets counts in each mouse. **(C)** Femurs from rAAV9-SIRT6-treated *LysMcre* mice were detected to identify EGFP-expressing cells. Scale bars = 50 µm. White arrow, $EGFP^{+}$ macrophages. **(D)** Macrophages were collected to detect SIRT6 protein with quantitative measurements at right. **(E)** Representative micro-CT images trabecular and cortical bone and quantitative measurements of Tb.BV/TV, Tb.N, Tb.Sp and Ct.Th in rAAV9-control- and rAAV9-SIRT6-injected *LysMcre* mice. **(F)** Dynamic histomorphometry of Tb and Eb and quantification of MAR, MS/BS and BFR/BS (n=6). Scale bars = 20 µm. **(G)** Immunofluorescence assay of $COL1^{+}$ area on trabecular and cortical bone surface. Scale bars: 100 µm. **(H)** Representative masson and **(I)** OCN staining images showing femoral trabecular bone mass and osteoblasts in rAAV9-control- and rAAV9-SIRT6-injected *LysMcre* mice. Scale bars = 50 µm. **(J)** Representative TRAP staining images of extraction socket in rAAV9-control- and rAAV9-SIRT6-injected *LysMcre* mice and quantification of osteoclasts per bone surface (n=6). Scale bar = 50 µm. **(K)** Quantification of apoptotic cell numbers on bone surface in rAAV9-control- and rAAV9-SIRT6-treated *LysMcre* mice. **(L)**

Quantification of SIRPα in F4/80$^+$ macrophages in rAAV9-control- and rAAV9-SIRT6-treated *LysMcre* mice. n = 6. Results are presented as the mean ± S.D. **p* < 0.05; #*p* > 0.05 by unpaired 2-tailed Student's t test.

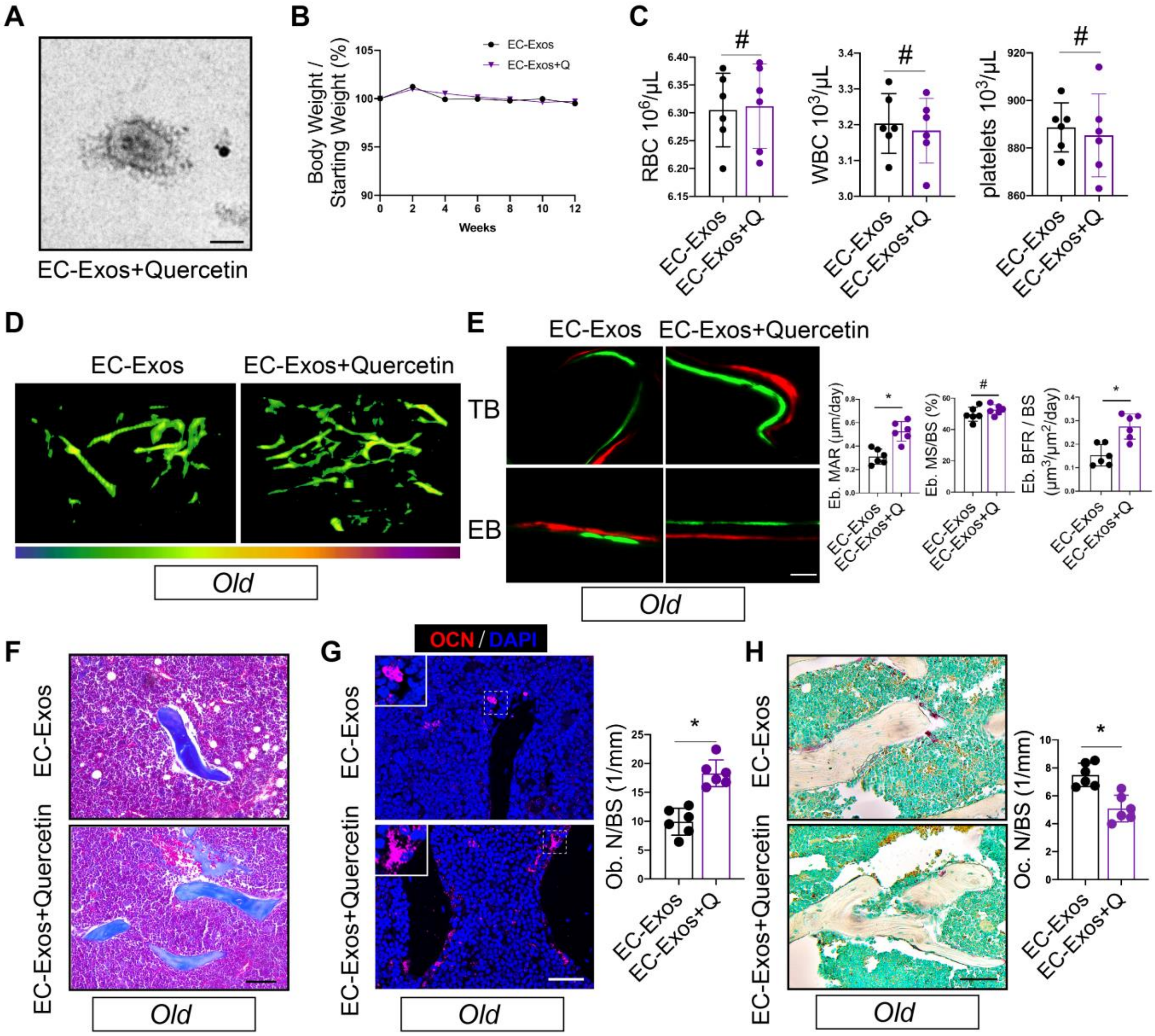

Supplementary Figure 12. EC-Exos loaded with quercetin effectively prevent bone loss. (A) Representative TEM image of quercetin loaded EC-Exos. Scale bars = 50 nm. **(B)** Normalized body weights in old mice treated with EC-Exos or quercetin-loaded EC-Exos. **(C)** Total white cell counts, red cell counts and platelets counts in each mouse. **(D)** The trabecular thickness of trabecular bone was color coded in old mice with injection of EC-Exos or quercetin loaded EC-Exos. **(E)** Representative images of dynamic histomorphometry of Tb and Eb in old mice with injection of EC-Exos or quercetin loaded EC-Exos. Scale bars = 20 μm. **(F)** Masson and **(G)** OCN staining showing femoral trabecular bone mass and osteoblasts in old mice with injection of EC-Exos or quercetin loaded EC-Exos. Scale bars = 100 μm **(F)**; 50 μm **(G)**. **(H)** Representative TRAP staining images of extraction socket in in old mice treated with EC-Exos or quercetin-loaded EC-Exos and quantification of osteoclasts per bone surface (n=6). Scale bar = 50 μm. Results are presented as the mean ± S.D. *p < 0.05; #p > 0.05 by unpaired 2-tailed Student's t test.

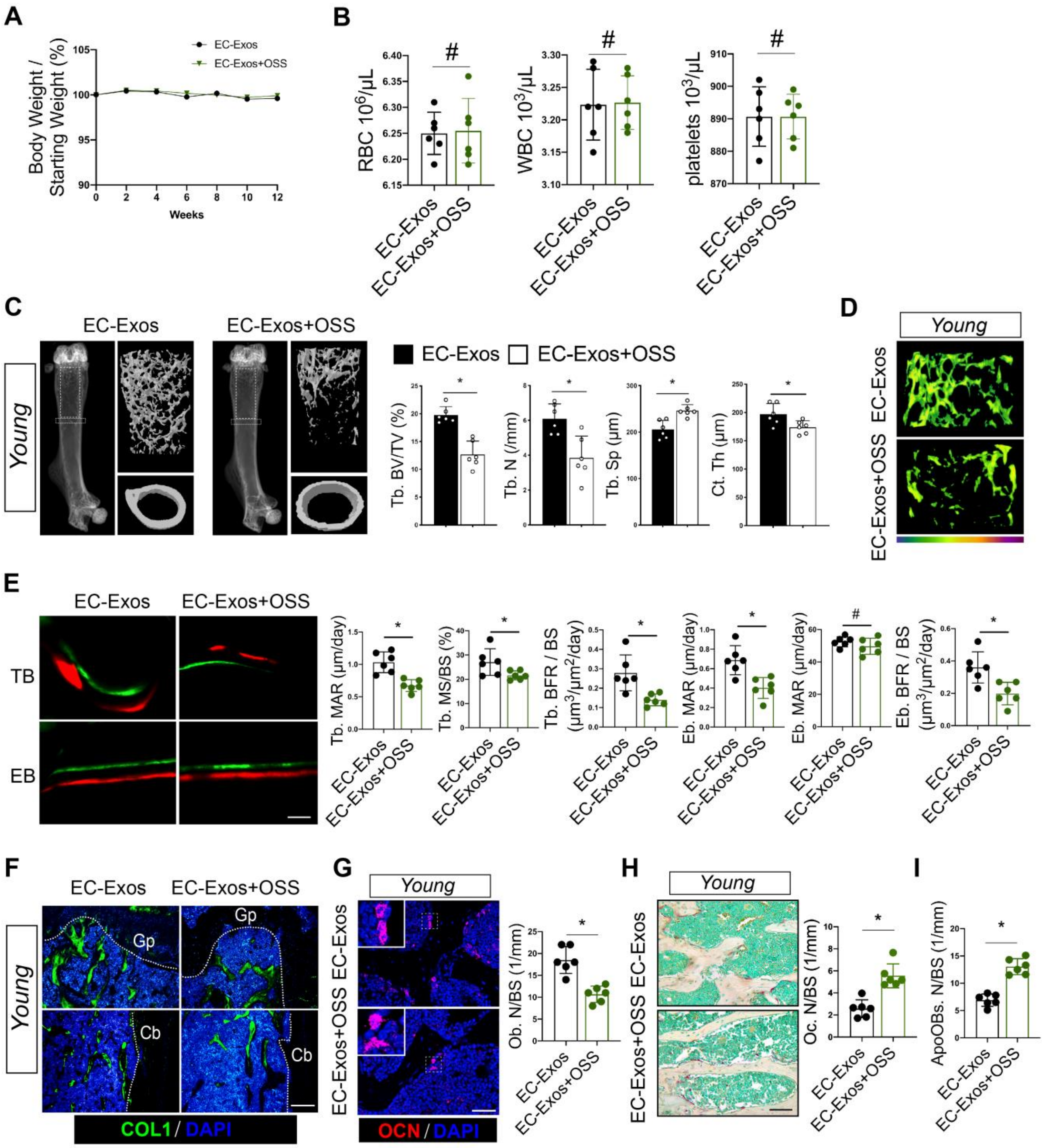

Supplementary Figure 13. EC-Exos loaded with OSS_128167 markedly deteriorate bone loss. (A) Normalized body weights between EC-Exos or OSS_128167 loaded EC-Exos injected young mice. OSS, OSS_128167. **(B)** Total white cell counts, red cell counts and platelets counts in each mouse (n=6). **(C)** Representative 3D reconstruction femoral trabecular bone mass and cortical bone thickness in EC-Exos or OSS_128167 loaded EC-Exos injected young mice. Quantitative measurements of Tb.BV/TV, Tb.N, Tb.Sp and Ct.Th. n=6. **(D)** The trabecular thickness of trabecular bone was color coded. **(E)** Representative images of dynamic histomorphometry of Tb and Eb in young mice with injection of EC-Exos or OSS_128167 loaded EC-Exos (n=6). Scale bars = 20 μm. **(F)** Immunofluorescence assay of COL1$^+$ area showing the difference of femoral trabecular bone mass. Scale bars = 200 μm. n=6. **(G)** OCN staining images showing the difference of osteoblasts on trabecular bone surface in young mice with injection of EC-Exos or OSS_128167 loaded EC-Exos (n=6). Scale bars = 50 μm. **(H)** Representative TRAP staining images of extraction socket in in EC-Exos or OSS_128167 loaded EC-Exos injected young mice and quantification of osteoclasts per bone surface

(n=6). Scale bar = 50 μm. **(I)** ApoOBs numbers in EC-Exos or OSS_128167 loaded EC-Exos injected young mice. n=6. Results are presented as the mean ± S.D. *$p < 0.05$; #$p > 0.05$ by unpaired 2-tailed Student's t test.

Supplementary Materials and Methods

Immunostaining

Cells were incubated on coverslips for 48 h at 37 °C, fixed with 4% paraformaldehyde and permeabilized in 1% Triton X100 for 10 min. Next, antigens were pre-incubated with goat serum to block nonspecific staining, and primary antibodies were incubated with cells overnight at 4 °C. Then, coverslips were rinsed with TBS, incubated with secondary antibody at 37 °C for 1 h and labeled with DAPI at room temperature for 1.5 min. Images were captured under fluorescence microscope in five random chosen fields from three independent samples (Leica Microsystems, Mannheim, Germany).

Dual luciferase assays

HER293T cells were seeded at the concentration of 5×10^5 in each well of 24-well plates. After 24 hours, these cells were co-transfected with pGL3-basic luciferase reporter vector, Renilla vector (pRL-TK; Promega, Madision, WI, USA), miRNA control, mimics or inhibitors using Lipofectamine 2000 (Invitrogen). For 48 hours incubation, luciferase activities were measured by using the Dual-Luciferase Reporter Assay System (Promega, Madision, WI, USA). Firefly luciferase activity was normalized to Renilla luciferase activity for each sample.

NanoLC-MS/MS analysis

LC-MS/MS analysis of apoOBs was performed in three replicates. For each sample, total peptides were harvested and measured with a nano-UPLC (EASY-nLC1200) coupled to a Q Exactive HFX Orbitrap instrument (Thermo Fisher Scientific) with a nano-electrospray ion source. Separation was loaded on a reversed-phase column (100 ID ×15 cm, Reprosi-Pur 120 C18-AQ, 1.9). Mobile phases were H2O with 0.1% FA, 2% ACN (phase A) and 80% ACN, 0.1% FA (phase B). Separation of sample was eluted with a 60 min gradient at 300 nL/min flow rate. Gradient B: 2-5% for 2 min, 5-22% for 44 min, 22-45% for 10 min, 45-95% for 2 min, 95% for 2 min. For MS1, data dependent acquisition (DDA) was conducted in profile and positive mode with Orbitrap analyzer at a resolution of 120,000 (@200 m/z) and m/z range of 350-1600; For MS2, the resolution was adjusted to 15,000 with a dynamic first mass. The automatic gain control (AGC) target for MS1 was set to 3E6 with max IT 50 ms, while 1E5 for MS2 with max IT 110 ms. The top 20 most intense ions were fragmented by HCD with normalized collision energy (NCE) of 27%, and isolation window of 1.2 m/z.

miRNA-sequencing (miRNA-seq)

For miRNA sequencing (miRNA-seq) of apoEVs, total RNA samples were reversed into indexed cDNA sequencing libraries using Small RNA Library Prep Kit (KAITAI-BIO). Firstly, the 3′ hydroxy of miRNAs were added with a single stranded adenylated DNA adapter using Small RNA 3 ADT. Subsequently, small RNA RT Primer was added to prevent ligation to the 5′ end. Then, a 5′ adapter was added to the 5′ phosphate using Small RNA 5 ADT and after adapter ligation, single stranded cDNA was obtained through a reverse transcription reaction. Next, cDNA was mixed with Small RNA index Primer using HANTAI SmallRNA Library Index Kit and performed for PCR amplification. The final libraries were then quantified and sequenced by KAPA Library Quantification Kit Illumina® Platforms (KAPA Biosystems).

Transfection, SA-β-gal and Trap staining

siRNA, miRNA inhibitors and mimics were synthesized by GenePharma Co. Ltd. (Shanghai, China). For

transient transfections, the siRNA, miRNA inhibitors, miRNA mimics, and negative control (50 nM, final concentration) were transfected into osteoblasts using Lipofectamine 2000 (Invitrogen, Carlsbad, CA, USA). Cells were performed for following experiments at least 48 h after transfection. The interference sequences used in this study are listed in Supplementary Table 8.
For SA-β-gal staining, osteoblasts and macrophages were seeded 12-well plates and stained with working solution according to SA-β-gal kit (Cat# GMS10012.1, GenMed). After 16 h incubation, five randomly fields were selected for quantification of SA β-gal positive cells under inverted microscopy. For Trap staining, monocyte-derived macrophages were further stimulated with RANKL (20 ng/ml, PeproTech) for 6 days to induce osteoclast differentiation and stained by tartrate-resistant acid phosphatase (TRAP) kit (Cat# N2250, Sigma-Aldrich). More than two positively stained nuclei were identified as matured osteoclasts.

Skeletal preparation and staining

The mice were skinned and eviscerated, and then skeletons were cleared in acetone for 48 h after fixed by 95% ethanol. Subsequently, the samples were stained with Alcian blue and Alizarin red solution, and finally immersed into 1% KOH until tissue had completely cleared.
